# Comparative Population Genomics and Machine Learning Reveal a Distinct Regional Genomic Fingerprint of Middle Eastern *Acinetobacter baumannii*

**DOI:** 10.3390/microorganisms14091971

**Published:** 2026-09-07

**Authors:** Blnd Azad Ismail, Areen Muhsin Abdulrahman, Shukur Wasman Smail

**Affiliations:** 1 Department of Biology, College of Science, Salahaddin University-Erbil, Erbil 44001, Kurdistan Region, Iraq; blndazad698@gmail.com (B.A.I.); erinamuhsin@gmail.com (A.M.A.); 2 Department of Genetics, Bio Diagnostic Center (BDC), Erbil 44001, Kurdistan Region, Iraq; 3 College of Pharmacy, Cihan University-Erbil, Erbil 44001, Kurdistan Region, Iraq; 4 Department of Medical Science, Respiratory Medicine, and Allergology, Uppsala University and University Hospital, 751 85 Uppsala, Sweden

**Keywords:** *Acinetobacter baumannii*, pangenome analysis, machine learning, Middle East, antimicrobial resistance, population genomics

## Abstract

*Acinetobacter baumannii* is a major multidrug-resistant nosocomial pathogen, yet its population structure and evolutionary dynamics in the Middle East remain poorly understood. This study compared Middle Eastern and global isolates to identify regional genomic signatures. We analyzed 199 high-quality *A. baumannii* genomes (100 Middle Eastern and 99 global) using pangenome reconstruction, core-genome phylogenetics, resistome and virulome profiling, genome-wide association studies, and machine learning. The pangenome comprised 10,971 genes, including 1102 unique to Middle Eastern isolates. Compared with global isolates, Middle Eastern genomes carried fewer antimicrobial resistance genes but a higher abundance of mobility-associated genes. A distinct regional cluster was dominated by sequence type 2 (ST2), representing 66% of Middle Eastern isolates. Genome-wide association analysis identified region-associated markers, particularly *ISAba1* variants and the *tufB* gene. A Random Forest classifier distinguished Middle Eastern from global isolates with 79.9% accuracy. Virulence gene repertoires were similar between groups, and resistance gene burdens remained stable over time. Middle Eastern *A. baumannii* isolates exhibit a distinct regional genomic signature characterized by accessory-genome conservation, reduced genomic heterogeneity, enrichment of *ISAba1*-associated markers, and increased mobility-associated gene content. These features differentiate the regional population from globally distributed isolates despite the similar prevalence of the ST2/IC2 lineage in both cohorts.

## 1. Introduction

*Acinetobacter baumannii* has become one of the most significant healthcare-associated pathogens worldwide. This Gram-negative opportunistic bacterium is implicated in a broad spectrum of infections, including ventilator-associated pneumonia, bloodstream infections, wound infections, and urinary tract infections, particularly among critically ill and immunocompromised individuals [1,2]. Its remarkable success as a nosocomial pathogen stems from a combination of biological adaptability, environmental resilience, and efficient transmission within healthcare environments [3]. Numerous studies have demonstrated that *A. baumannii* can persist for extended periods on hospital surfaces and medical devices, withstand desiccation and disinfectant exposure, and form robust biofilms on both biotic and abiotic surfaces, all of which facilitate its long-term survival and dissemination within healthcare settings [4,5].

The clinical success of *A. baumannii* is further enhanced by a diverse repertoire of virulence-associated factors, including Csu pili, biofilm-associated proteins, outer membrane protein A (OmpA), lipopolysaccharides, capsular polysaccharides, secretion systems, and iron acquisition mechanisms. Together, these determinants contribute to adhesion, immune evasion, host colonization, and survival under hostile environmental conditions [6]. Equally concerning is the organism’s extraordinary ability to acquire antimicrobial resistance (AMR) determinants, leading to the emergence of multidrug-resistant (MDR), extensively drug-resistant (XDR), and even pandrug-resistant strains. As a result, carbapenem-resistant *A. baumannii* has been designated a critical-priority pathogen by the World Health Organization and is now regarded as a major threat to global public health [7].

A defining feature of the evolutionary success of *A. baumannii* is its exceptional genomic plasticity. Comparative genomic studies have revealed that the species possesses an open pangenome consisting of a relatively conserved core genome alongside a highly dynamic accessory genome that continues to expand through horizontal gene transfer (HGT) [8,9]. Mobile genetic elements (MGE), including plasmids, transposons, insertion sequences, integrons, and genomic islands, play a fundamental role in this process by promoting the acquisition and dissemination of AMR genes and other adaptive genetic traits [10]. Furthermore, recombination events, gene acquisition and loss, and insertion-sequence-mediated genomic rearrangements contribute substantially to the rapid evolutionary capacity of the species, enabling adaptation to a wide range of ecological niches and clinical environments [2,6].

The advent of whole-genome sequencing has substantially advanced our understanding of the epidemiology and population structure of *A. baumannii*. Population genomic investigations have shown that the global dissemination of this pathogen is largely driven by a limited number of highly successful international clones, particularly International Clone II (IC2), which predominates in healthcare settings worldwide [11]. Large-scale genomic studies have demonstrated that transmission is shaped by a combination of local clonal expansion, repeated introductions, and ongoing HGT, generating genetically diverse populations influenced by both regional and global evolutionary pressures [9,12]. In addition, genomic evidence indicates that clinically relevant lineages may circulate beyond hospital environments and can involve environmental, community, animal, and food-associated reservoirs, emphasizing the importance of a One Health perspective for understanding the ecology and spread of this pathogen [13].

The burden of MDR *A. baumannii* is particularly severe in the Middle East, where numerous studies have reported high rates of multidrug resistance and carbapenem resistance among clinical isolates [14,15]. The pathogen is especially prevalent in intensive care units, where prolonged hospitalization, invasive procedures, mechanical ventilation, and extensive antimicrobial exposure facilitate the selection and transmission of resistant clones [15]. Reports from Saudi Arabia, Jordan, Iran, Iraq, and other countries consistently highlight the substantial clinical impact of MDR *A. baumannii*, including increased mortality, extended hospital stays, and restricted therapeutic options [12,13]. Genomic studies conducted across the region have identified IC2 as the predominant lineage, with sequence type 2 (ST2) and closely related sequence types such as ST208 and ST195 repeatedly reported in multiple countries [15,16]. Moreover, emerging evidence suggests that ST158 may represent a lineage that is particularly enriched within the Middle East and neighboring geographical regions [17]. 

The clinical burden of MDR *A. baumannii* has become an increasing concern across the Middle East, with high levels of AMR reported in diverse healthcare settings. In Lebanon, a five-year study (2017–2021) at the Geitaoui–University Medical Center documented a substantial burden of *A. baumannii* and AMR, highlighting the persistent challenge of managing these infections in regional tertiary-care settings [18]. Similarly, studies from Baghdad, Iraq, have reported high prevalence of MDR and carbapenem-resistant *A. baumannii* among clinical specimens, indicating a substantial regional burden of AMR [19]. In Egypt, molecular characterization of carbapenem-resistant *A. baumannii* clinical isolates has further demonstrated the presence of diverse resistance determinants among affected patients [20]. Collectively, these findings highlight the substantial burden of MDR and carbapenem-resistant *A. baumannii* in Middle Eastern healthcare settings and underscore the need for continued genomic surveillance to better understand the population structure, resistance determinants, and regional dissemination of high-risk *A. baumannii* lineages.

Despite the increasing availability of genomic data, substantial gaps remain in our understanding of the population structure and evolutionary dynamics of Middle Eastern *A. baumannii* populations. Most published studies are based on single institutions, relatively small isolate collections, or short sampling periods, limiting the ability to investigate long-term evolutionary processes, regional dissemination patterns, and cross-border transmission events [12,13]. Furthermore, genomic surveillance efforts remain geographically uneven, and only a limited number of studies have combined pangenome analyses, mobile genetic element characterization, environmental surveillance, and longitudinal epidemiological data within a comprehensive analytical framework [21]. Consequently, the extent to which Middle Eastern populations possess distinct genomic characteristics that differentiate them from globally distributed isolates remains largely unresolved.

Recent developments in machine learning have created new opportunities for analyzing increasingly complex genomic datasets. Machine learning methodologies have been successfully applied to AMR prediction, lineage assignment, biomarker discovery, and the identification of previously unrecognized genomic signatures within bacterial populations [12,13,15]. Although several studies have demonstrated the capacity of machine learning algorithms to detect geographically structured and environment-associated genomic patterns, their application to the identification of region-specific genomic signatures in *A. baumannii* remains relatively limited [22]. Consequently, the integration of comparative genomics with machine learning represents a promising approach for uncovering previously unrecognized patterns of regional adaptation and population differentiation.

Despite the recognized status of the Middle East as a global hotspot for MDR *A. baumannii*, the genomic characteristics and evolutionary dynamics of regional populations remain incompletely understood. To address this knowledge gap, we conducted a comparative population genomic analysis of 199 high-quality genomes representing Middle Eastern and globally distributed isolates. The primary aim of this study was to identify genomic features that distinguish Middle Eastern *A. baumannii* populations from those circulating worldwide and to assess the role of clonal expansion in shaping regional genomic diversity. In addition, we sought to characterize population structure, AMR determinants, virulence-associated factors, and pangenome composition, while evaluating the potential of machine learning approaches to identify and validate region-specific genomic signatures. Collectively, this work provides new insights into the evolution and regional adaptation of *A. baumannii* and establishes a framework for future genomic surveillance and antimicrobial stewardship efforts. However, previous studies have largely focused on individual countries, hospital outbreaks, specific resistance determinants, or individual epidemic lineages, and a comparative genomic assessment of the broader Middle Eastern *A. baumannii* population against a geographically diverse global background remains limited. In this study, we integrate pangenome analysis, core-genome phylogeny, resistome and virulome profiling, pangenome-wide association study (GWAS), temporal analysis, and machine-learning approaches to characterize the genomic structure of Middle Eastern *A. baumannii* in comparison with globally distributed isolates. Beyond identifying predominant sequence types and resistance determinants, we investigate whether these features collectively define a distinct regional genomic pattern and assess its potential implications for regional surveillance and infection prevention and control.

## 2. Materials and Methods

### 2.1. Comprehensive Genome Acquisition and Stratification

A total of 200 *Acinetobacter baumannii* whole-genome assemblies were retrieved from the National Center for Biotechnology Information (NCBI) database using the NCBI Datasets command-line interface (CLI; v16.2) (https://ftp.ncbi.nlm.nih.gov/pub/datasets/, accessed on 12 April 2026). Genome selection was performed using a predefined multi-stage filtration strategy to ensure dataset consistency, minimize assembly-related bias, and enable a meaningful comparison between Middle Eastern and globally distributed isolates. Inclusion criteria were defined as follows: (i) confirmed human host origin; (ii) availability of reliable geographic metadata at the country level; (iii) availability of the year of isolation within the associated metadata; and (iv) assignment to the *A. baumannii* species. The final dataset (*n* = 199) spanned a temporal range from 1948 to 2026. The Middle Eastern cohort (*n* = 100) comprised isolates collected between 2008 and 2016. The Global cohort (*n* = 99) included isolates collected between 2003 and 2026, including one historical isolate from the United States collected in 1948. All remaining global isolates (*n* = 98) were collected between 2003 and 2026.

**Figure 1:**
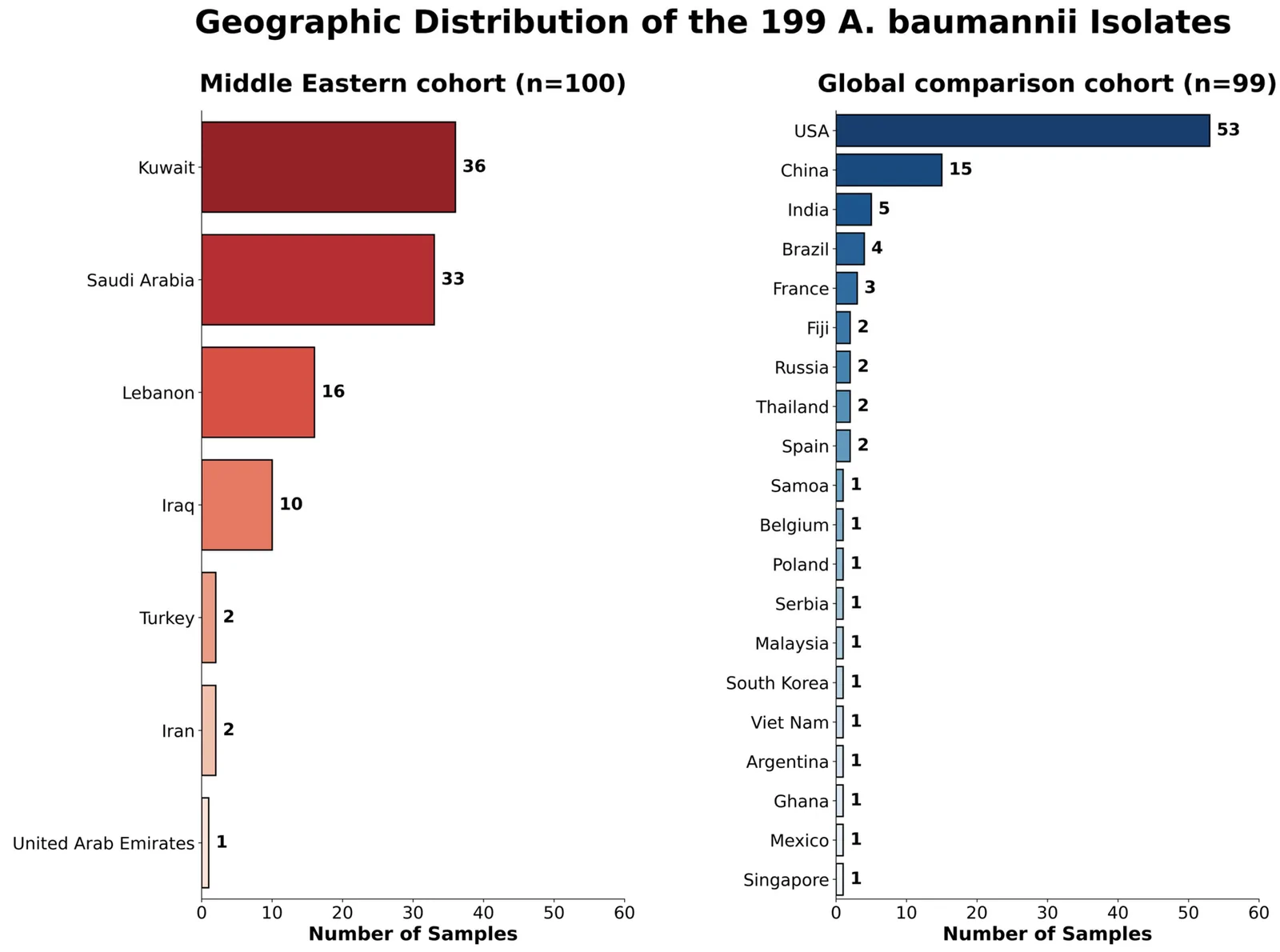
Geographic distribution of the 199 *A. baumannii* isolates included in the study. The figure shows the country-level distribution of isolates across the Middle Eastern and Global comparison cohorts. The Middle Eastern cohort comprised 100 isolates from seven countries, with the largest contributions from Kuwait (*n* = 36), Saudi Arabia (*n* = 33), Lebanon (*n* = 16), and Iraq (*n* = 10), followed by Turkey (*n* = 2), Iran (*n* = 2), and the United Arab Emirates (*n* = 1). The Global comparison cohort comprised 99 isolates distributed across countries representing Africa, Asia, Europe, North America, Oceania, and South America, with the largest contributions from the USA and China, followed by India, Brazil, France, and other countries with smaller numbers of isolates. *A. baumannii*, *Acinetobacter baumannii*; *n*, sample size; USA, United States of America.

The selected genomes were stratified into two cohorts. The Middle Eastern cohort (*n* = 100) comprised isolates from the United Arab Emirates, Lebanon, Saudi Arabia, Turkey, Iran, Iraq, and Kuwait, whereas the Global comparison cohort (*n* = 100) comprised isolates representing a geographically diverse distribution across, Africa, Asia, Europe, North America, Oceania and South America. This stratification was designed to provide a balanced regional comparison while maintaining broad geographic representation in the global cohort, as summarized in Figure 1.

Exclusion criteria were applied during the initial screening to minimize the inclusion of genomes that did not meet the predefined selection requirements. Genomes were excluded if they originated from non-human hosts, lacked country-level geographic information or isolation-year metadata, or did not meet the predefined assembly-quality criteria described below. Following the initial screening and quality assessment, one assembly from the Global cohort was excluded because of excessive assembly fragmentation (N50 < 10,000 bp). Consequently, the final dataset consisted of 199 genomes, comprising 100 Middle Eastern isolates and 99 Global isolates, which were used for all subsequent comparative genomic, phylogenetic, and pangenome analyses. To ensure consistency and facilitate downstream data management, all retained FASTA files were renamed according to a standardized naming convention in the format Region_Country_Accession_Year.fasta.

### 2.2. Standardized Genome Annotation and Assembly Quality Assessment

To minimize inconsistencies arising from the use of different annotation pipelines and database versions in public NCBI records, all 199 retained *A. baumannii* genomes were subjected to standardized de novo genome annotation using Prokka v1.14.6. [23]. Annotation was performed using the genus- and species-specific parameters—genus *Acinetobacter* and—species *baumannii*, respectively. A minimum contig length of 200 bp was applied during annotation to exclude very short contigs that may represent assembly artifacts or unreliable genomic fragments. The standardized annotation procedure enabled consistent identification and classification of coding sequences (CDSs), ribosomal RNA (rRNA) genes, transfer RNA (tRNA) genes, and other genomic features across all isolates.

Following annotation, the structural quality of the genome assemblies was assessed using QUAST v5.0.2. [24]. Quality-control assessment included evaluation of total assembly length, number of contigs, N50, and total CDS counts generated during the standardized annotation process. Assemblies with a total genome size outside the predefined *A. baumannii* range of 3.7–4.2 Mbp, more than 500 contigs, or an N50 < 10,000 bp were considered excessively fragmented or outside the predefined quality criteria. The assembly-quality assessment was used to verify the consistency of the retained genomes and to identify potential abnormalities that could influence downstream pangenome reconstruction, phylogenetic analysis, and comparative genomic analyses. The use of a uniform annotation pipeline and standardized quality-control criteria across all 199 genomes reduced technical variation attributable to differences in public database annotation procedures and provided a consistent genomic feature set for subsequent analyses.

### 2.3. Pangenome Reconstruction and Hierarchical Clustering

The pangenome of the 199 *A. baumannii* genomes was reconstructed using Panaroo v1.3.0. [25]. To minimize the inclusion of assembly artifacts, fragmented gene calls, and spurious annotations, Panaroo was executed using the strict cleaning mode (–clean-mode strict) together with the –remove-invalid-genes option. Gene clustering was performed using a 95% nucleotide/protein sequence identity threshold to group homologous gene sequences into pangenome gene families. The resulting gene presence–absence matrix was used for subsequent pangenome characterization and comparative analyses. Pangenome gene families were classified according to their frequency across the 199 genomes. Core genes were defined as genes present in ≥99% of genomes, soft-core genes as those present in 95–<99%, shell genes as those present in 15–<95%, and cloud genes as those present in <15% of genomes. This classification enabled differentiation between highly conserved genomic components and variable accessory gene fractions.

To investigate genomic relatedness and regional variation, the binary gene presence–absence matrix was used to calculate a Jaccard distance matrix, which measures dissimilarity based on shared and non-shared gene content and is therefore appropriate for binary pangenomic data. Hierarchical clustering was subsequently performed using the complete-linkage method. Clustering and heatmap visualization were implemented in Python 3.11 using SciPy v1.11 [26] and Seaborn v0.12. [26]. The resulting dendrogram and gene-content patterns were used to assess the degree of genomic similarity among isolates and to examine whether isolates from the Middle Eastern and Global cohorts formed distinct or overlapping genomic clusters. To further characterize the major patterns of variation in accessory gene content, Principal Component Analysis (PCA) was performed on the binary gene presence–absence matrix using Scikit-learn v1.3. [27]. PCA was used to reduce the dimensionality of the pangenomic dataset and visualize the major axes of variation among isolates. The resulting principal-component distributions were examined to assess the overall structure and separation of the Middle Eastern and Global cohorts.

### 2.4. Core-Genome Phylogenetic Analysis

The evolutionary relationships among the 199 *A. baumannii* isolates were inferred using a high-resolution core-genome phylogenetic analysis. The multiple sequence alignment (MSA) of the filtered core genes identified by Panaroo v1.3.0 was used as the input for phylogenetic reconstruction. Maximum-likelihood (ML) analysis was performed using IQ-TREE 2 v2.2.0 [28]. Under the General Time Reversible model with gamma-distributed rate variation (GTR+G), which accounts for differences in nucleotide substitution rates among sites. The statistical robustness of the inferred phylogenetic relationships was assessed using 1000 ultrafast bootstrap replicates (−bb 1000). The resulting ML tree was examined based on the bootstrap support values assigned to individual branches. The final phylogenetic tree was visualized and annotated using the Interactive Tree of Life (iTOL v6) [29], with geographic and cohort metadata incorporated to facilitate assessment of regional clustering patterns. Phandango v1.1.0 [30] was additionally used for interactive visualization of accessory genome variation alongside the core-genome phylogeny, enabling comparison of phylogenetic relationships with patterns of gene presence–absence across the isolates.

### 2.5. Genotypic Profiling of AMR, Virulence, and Sequence Types

The genomic determinants associated with AMR, virulence, and clonal population structure were systematically characterized across all 199 *A. baumannii* isolates. AMR-associated genes were identified by screening the annotated genome sequences against the Comprehensive Antibiotic Resistance Database (CARD) using Abricate v1.0.1. [31]. Virulence-associated genes were similarly identified by screening against the Virulence Factor Database (VFDB) [32] using Abricate. For both AMR and virulence profiling, matches were retained as high-confidence hits when they met minimum thresholds of ≥80% sequence identity and ≥80% gene coverage. These criteria were applied to minimize the inclusion of weak or partial sequence matches and to improve the reliability of downstream comparative analyses. To characterize the population structure and identify clonal lineages, multilocus sequence typing (MLST) was performed using the mlst tool v2.23. [33]. Sequence types were assigned according to the available Oxford and Pasteur MLST schemes for *A. baumannii*. The resulting ST profiles were used to assess the distribution of major clonal lineages across the Middle Eastern and Global cohorts and to examine their relationship with the observed patterns of AMR, virulence-associated genes, and core-genome phylogeny.

### 2.6. Evolutionary Trajectory and Statistical Association Analyses

Mobility-associated gene abundance was quantified from the Prokka-generated GFF files by counting individual genes annotated as transposases or integrases in each isolate. Each annotated copy was counted independently, including multiple copies of the same mobility-associated gene within a genome. For each isolate, the total number of transposase and integrase annotations was used as the mobility-associated gene count. Group-level abundance was calculated as the mean number of these annotations per isolate across the 100 Middle Eastern and 99 global genomes, yielding mean values of 20.42 and 17.28, respectively. This metric represents the abundance of genes associated with genome mobility and was used as a proxy for genomic plasticity; it does not constitute a structural or comprehensive measurement of the mobilome. These analyses were performed to investigate temporal trends in resistance gene acquisition and mobility-associated gene abundance within the study population. Associations between accessory genes and geographic origin were assessed using Scoary v1.6.16. [34]. Genes associated with the Middle Eastern cohort were evaluated using Bonferroni-adjusted *p*-values (<0.05) and odds ratios (ORs). To account for potential population-structure effects, the pairwise comparison algorithm with 1000 permutations was applied. These analyses were used to identify accessory genomic features associated with regional origin.

To further evaluate the discriminatory power of the region-associated genetic markers, a Random Forest classifier was implemented using Scikit-learn. The model was trained using the pangenome gene presence–absence matrix to classify isolates according to geographic origin (Middle East vs. Global). The classifier used 100 estimators (n_estimators = 100) and was evaluated using 5-fold stratified cross-validation. Feature importance was assessed using Gini importance as implemented in Scikit-learn v1.3.0 to identify genomic markers contributing most strongly to regional classification.

### 2.7. Secondary Functional Characterization of the Hypothetical Proteome

To investigate the biological significance of the 978 Middle East-specific accessory genes initially annotated as hypothetical proteins, a secondary conserved-domain characterization was performed. Protein sequences were extracted from the 199-genome dataset. To minimize the influence of potential annotation artifacts and fragmented open reading frames (ORFs), sequences were filtered for completeness based on the presence of valid start and stop codons. A total of 915 high-quality protein sequences were retained for secondary characterization, while 63 sequences were excluded during this validation step. Conserved domains were identified using the NCBI Batch Conserved Domain Search (CD-Search) (https://www.ncbi.nlm.nih.gov/Structure/bwrpsb/bwrpsb.cgi, accessed on 12 August 2026) (R2–4) against the Conserved Domain Database (CDD) v3.20, using an E-value threshold of 0.01. The identified domains were subsequently classified according to their functional associations, including metabolic/enzymatic functions, prophage-associated functions, mobile DNA and plasmid stability, persistence and toxin–antitoxin systems, stress response and DNA repair, and transcriptional regulation.

### 2.8. Statistical Analysis

Statistical associations between individual accessory gene families and geographic origin were determined using a GWAS framework implemented in Scoary v1.6.16. Associations were evaluated across the 10,971-gene pangenome using nominal *p*-values derived from Fisher’s exact test. To account for multiple testing and reduce the risk of false-positive associations (Type I errors), Bonferroni correction was applied to the *p*-values obtained for individual gene families; statistical significance was defined as a Bonferroni-adjusted *p* < 0.05. The OR was used to quantify the strength and direction of association between individual genes and geographic origin, with an OR > 1.0 indicating a positive association with the Middle Eastern cohort and an OR < 1.0 indicating a relative association with the Global cohort. The OR was considered in conjunction with the adjusted *p*-value when interpreting regional genetic markers. Furthermore, the stability of the identified gene–origin associations was evaluated using 1000 random permutations to assess the robustness of the observed associations against random reassignment of cohort labels.

## 3. Results

### 3.1. Pangenome Composition and Accessory Genome Diversity

A total of 199 *A. baumannii* genomes, comprising both Middle Eastern and globally distributed isolates, were subjected to a comprehensive comparative genomic analysis. This study employed a multi-faceted analytical pipeline, including pangenome characterization, resistance and mobilome profiling, hierarchical clustering of accessory genes, PCA, and GWAS. Furthermore, temporal mobilome dynamics, virulence factor profiling, and the distribution of specific resistance determinants were evaluated. These analyses identified fundamental divergences in genomic architecture, accessory gene composition, and resistance-associated markers, revealing distinct regional clustering patterns that differentiate Middle Eastern isolates from the global population.

**Figure 2:**
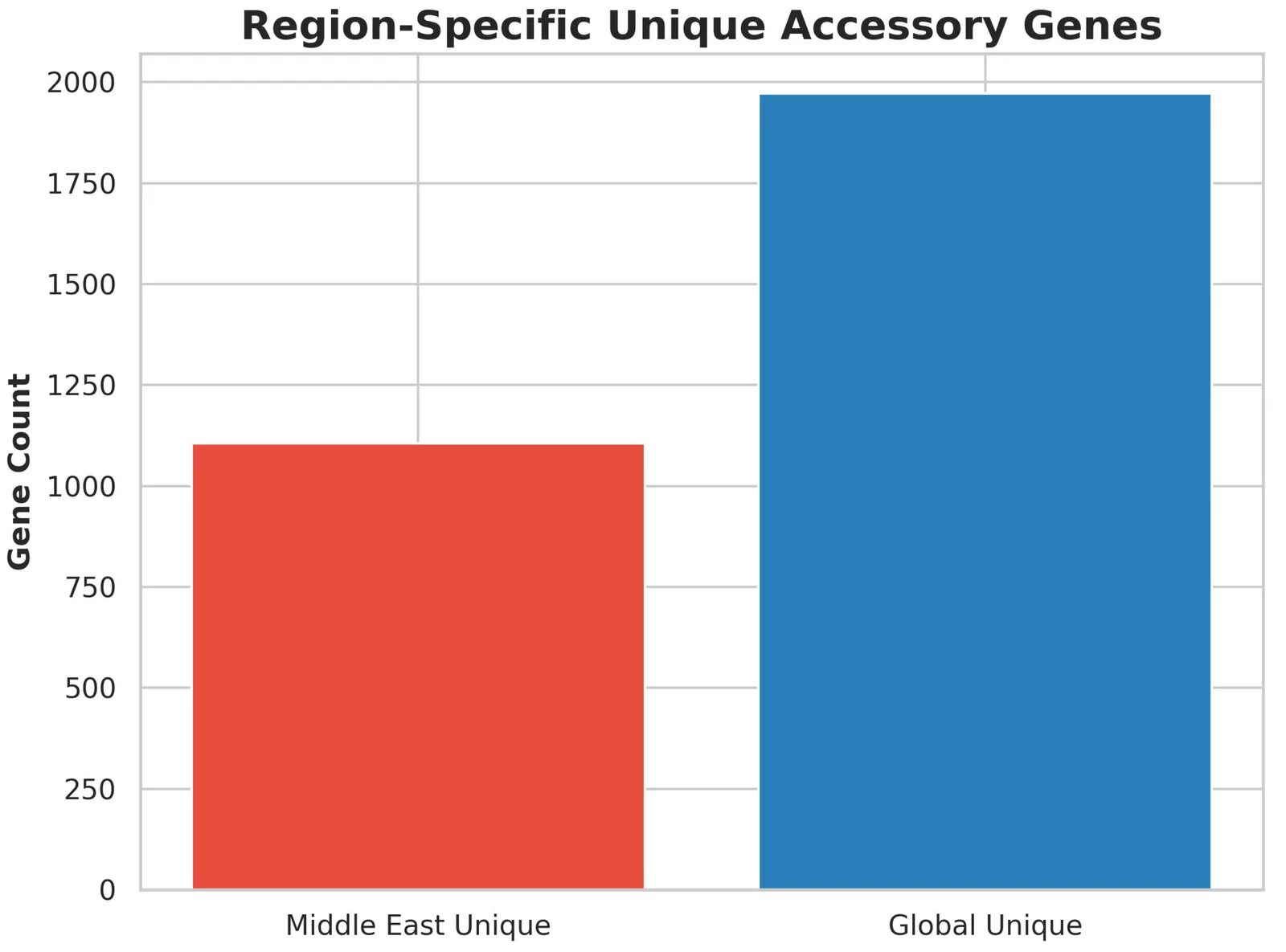
Region-specific accessory gene content in Middle Eastern and global *A. baumannii* isolates. The number of accessory genes unique to each geographic population is shown. Middle Eastern isolates contained 1102 unique genes, whereas the global population contained 1970 unique genes, demonstrating greater accessory genome diversity within the global dataset.

Pangenome analysis identified a total of 10,971 non-redundant genes across the dataset (Table 1). The core genome, consisting of 1495 genes, accounted for 13.6% of the total pangenome, whereas the accessory genome constituted the vast majority of the genetic repertoire. The cloud genome (defined as genes present in <15% of isolates) emerged as the most expansive component, containing 6687 genes (61.0%), followed by shell genes (15–95% prevalence) distributed at intermediate frequencies. Notably, the accessory genome exhibited significantly higher diversity than the conserved core. Regional comparisons further identified 1102 genes unique to Middle Eastern isolates and 1970 genes exclusive to the global collection (Figure 2). These region-specific genes were primarily localized within the accessory and cloud genome fractions rather than the core, indicating they are present only in restricted subsets of isolates and absent from the broader population.

### 3.2. Comparative Antimicrobial Resistance Gene Burden and Mobilome Profiles

Assessments of resistance gene burden and mobilome content revealed diverging regional profiles (Table 2). Global isolates possessed a higher average number of resistance genes (37.63 ± 9.67) compared to Middle Eastern isolates (30.09 ± 4.53). The resistance gene count among global isolates showed significantly greater variability, as evidenced by a standard deviation (9.67) that is more than double that of the Middle Eastern group (4.53). This indicates that global genomes exhibit a wider range of resistance profiles, with several genomes possessing markedly elevated gene counts relative to the mean. Conversely, the lower standard deviation in Middle Eastern isolates suggests a more constrained distribution and higher genomic homogeneity regarding resistance within the regional population.

In contrast to the resistance gene burden, Middle Eastern isolates exhibited a higher abundance of mobility-associated genes, with a mean mobilome value of 20.42 ± 17.87, compared to 17.28 ± 20.72 in global isolates. While the Middle Eastern isolates had a higher mean, both groups displayed very high standard deviations relative to their means, indicating significant diversity in mobilome content across all samples. The global population showed the highest level of dispersion with a standard deviation of 20.72, reflecting a broad variance that includes several highly mobile outlier genomes. Although the Middle Eastern cohort also exhibited a wide distribution (standard deviation of 17.87), the spread was slightly less pronounced than the global set. Consequently, the regional comparison highlighted an inverse relationship between resistance gene burden and mobility-associated gene abundance when comparing these two populations.

### 3.3. Functional Characterization of Middle East-Specific Accessory Genes

**Figure 3:**
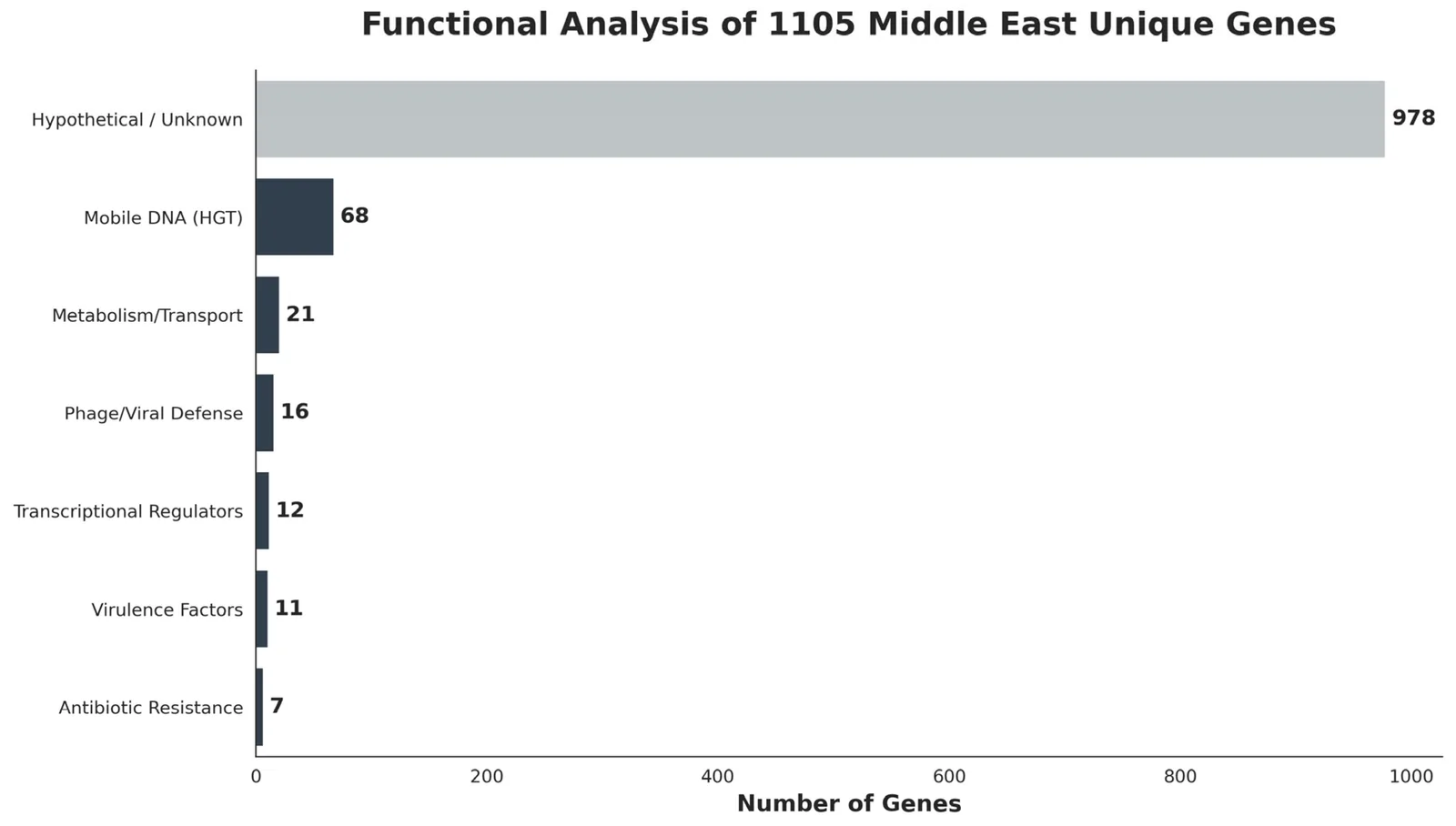
Functional classification of Middle East-specific accessory genes. Functional annotation of 1102 genes unique to Middle Eastern *A. baumannii* isolates. Hypothetical proteins constituted the largest category (978 genes), followed by genes associated with MGE and HGT (68 genes). Smaller functional groups included metabolism/transport, phage defense systems, transcriptional regulators, virulence factors, and antibiotic resistance genes. The predominance of hypothetical proteins indicates extensive uncharacterized genomic diversity within the Middle Eastern accessory genome.

Functional annotation of the Middle East-specific accessory genome is summarized in Figure 3. The initial annotation identified HGT- and MGE-associated genes as the most prominent characterized category, including approximately 68 transposases, integrases, insertion sequences, and recombination-associated proteins, followed by 16 phage-defense genes and seven resistance-associated determinants. Notably, 978 of the 1102 Middle East-specific genes (88.9%) were initially annotated as hypothetical proteins. Secondary conserved-domain characterization of the 915 validated sequences identified recognizable domains in 424 (46.3%), with most associated with metabolic or enzymatic functions (358, 84.4%), followed by prophage-associated functions (29, 6.8%), mobile DNA and plasmid stability (16, 3.8%), persistence and toxin–antitoxin systems (8, 1.9%), stress response and DNA repair (8, 1.9%), and transcriptional regulation (5, 1.2%) (Table 3). The remaining 491 proteins lacked recognizable conserved domains and remained functionally unresolved.

This functional profile was also characterized by a high proportion of hypothetical proteins, which accounted for approximately 85% of Middle East-specific genes. As illustrated in the functional distribution, these uncharacterized proteins significantly outnumbered all known functional categories combined. Other minor functional groups included genes involved in transport systems, metabolic pathways, transcriptional regulation, and membrane- or DNA-associated proteins. Overall, HGT-related functions were the most abundant characterized features within the Middle Eastern-specific accessory genome.

### 3.4. Population Structure Based on Accessory Genome Analysis

**Figure 4:**
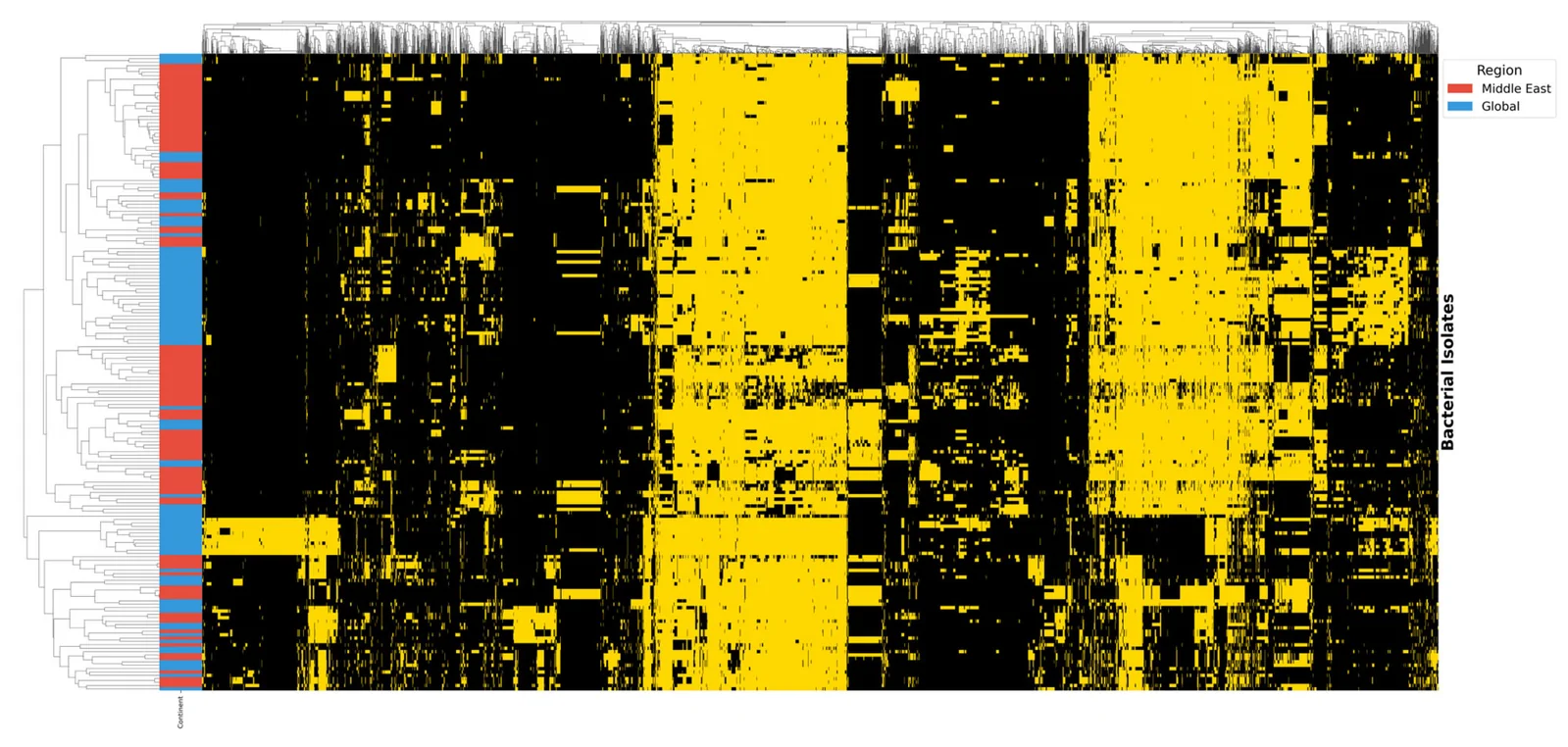
Hierarchical clustering of accessory genome content among Middle Eastern and global *A. baumannii* isolates. Hierarchical clustering based on the presence–absence patterns of 3354 accessory and shell genes. Rows represent isolates and columns represent genes. Gene presence is shown in dark yellow and absence in black. The sidebar indicates geographic origin (red, Middle East; blue, global). Middle Eastern isolates formed distinct clusters characterized by conserved accessory gene blocks, whereas global isolates exhibited more fragmented patterns, indicating greater accessory genome diversity and heterogeneity. This clustering demonstrates clear regional differentiation in accessory genome composition.

Hierarchical clustering of 3354 accessory and shell genes provided further insight into population structure (Figure 4). In this analysis, geographic origin was delineated by a color sidebar (red for Middle Eastern and blue for global isolates), with gene presence indicated in dark yellow. The resulting clustermap demonstrated a clear regional partitioning. Middle Eastern isolates primarily organized into three high-density clusters defined by continuous blocks of conserved accessory genes. These distinct vertical bands suggest a highly stable accessory genome shared across the Middle Eastern regional cohort. By contrast, global isolates exhibited substantial heterogeneity.

**Figure 5:**
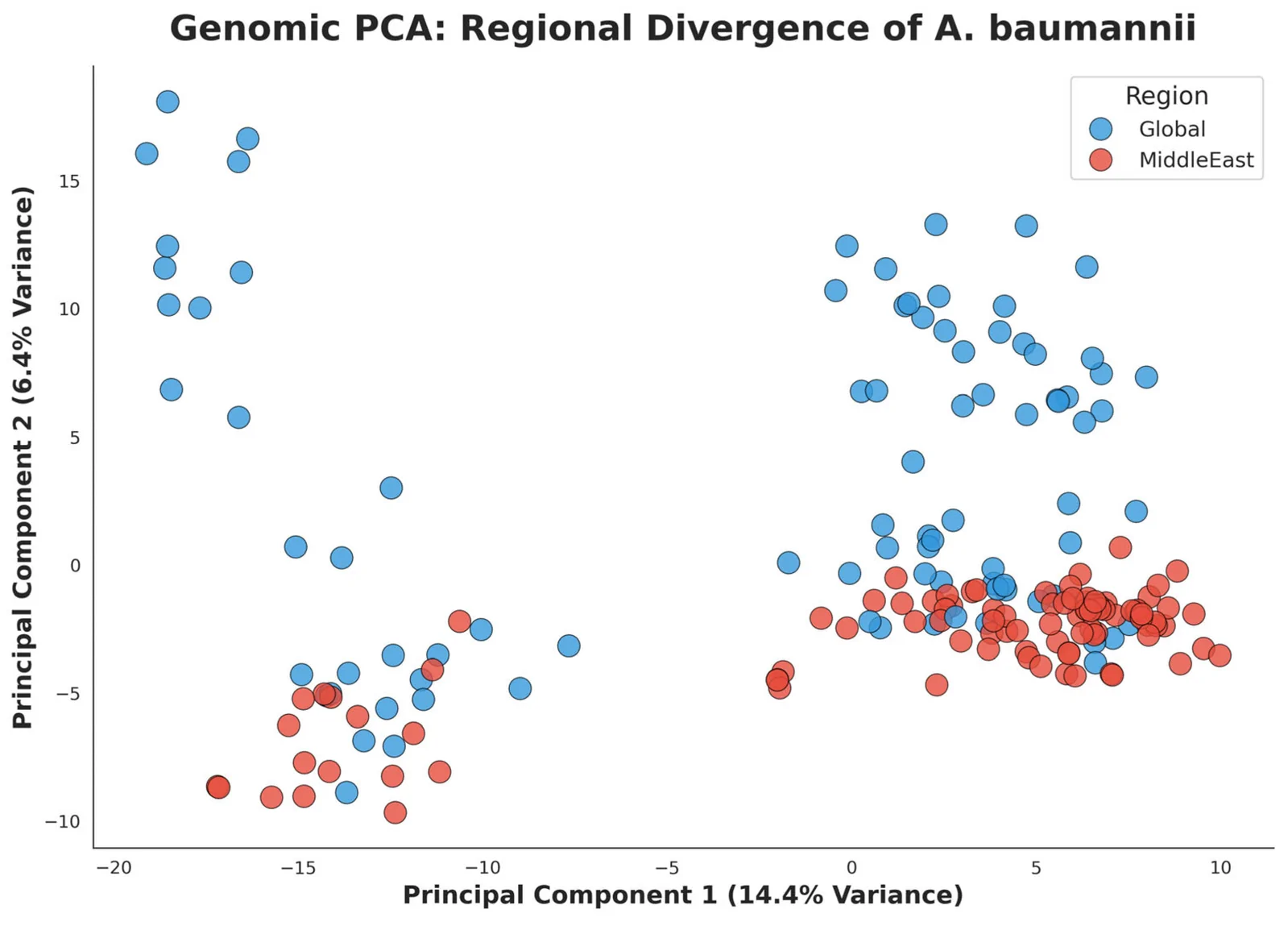
PCA of accessory genome variation among Middle Eastern and global *A. baumannii* isolates, PCA was performed using accessory genome profiles to assess population structure. Each point represents an individual isolate, colored according to geographic origin (red, Middle East; blue, global). PC1 explained 14.4% of the total variance, while Principal Component 2 (PC2) explained 6.4%. Middle Eastern isolates formed a compact cluster with limited dispersion, whereas global isolates were distributed more broadly across the PCA space, indicating greater genomic diversity. The tight clustering of Middle Eastern isolates is consistent with the predominance of ST2 within the regional population and supports the presence of distinct regional genomic signatures. PCA, principal component analysis; *A. baumannii*, *Acinetobacter baumannii*; %, percentage.

The global partition of the clustermap was characterized by fragmented and discontinuous patterns of gene presence and absence. Unlike the Middle Eastern isolates, the global population lacked large, conserved vertical blocks, showing instead frequent transitions in accessory gene content between neighboring isolates. This suggests that while several accessory gene blocks are repeatedly maintained across Middle Eastern clusters, the global accessory genome is significantly more scattered and variable. Sharp transitions between gene presence and absence further underscore the distinct segregation patterns between these geographic groups. PCA of the accessory genome corroborated these findings (Figure 5). The first principal component (PC1) explained 14.4% of the total variance and effectively separated the two populations along the horizontal axis. Middle Eastern isolates formed a dense, tightly grouped cluster in the lower right quadrant of the plot, reflecting minimal genomic dispersion. A secondary, smaller Middle Eastern cluster was observed in the lower left quadrant, though it exhibited slightly more spatial dispersion than the primary group.

Global isolates were distributed extensively across both the PC1 and PC2 axes, occupying a much larger area of the PCA space than their Middle Eastern counterparts. This population showed significant vertical dispersion and included several distant outliers. The PCA results thus reinforce the observation of reduced genomic variability within the Middle Eastern population relative to the global collection.

### 3.5. Genome-Wide Association Analysis Identifies Regional Genomic Markers

**Figure 6:**
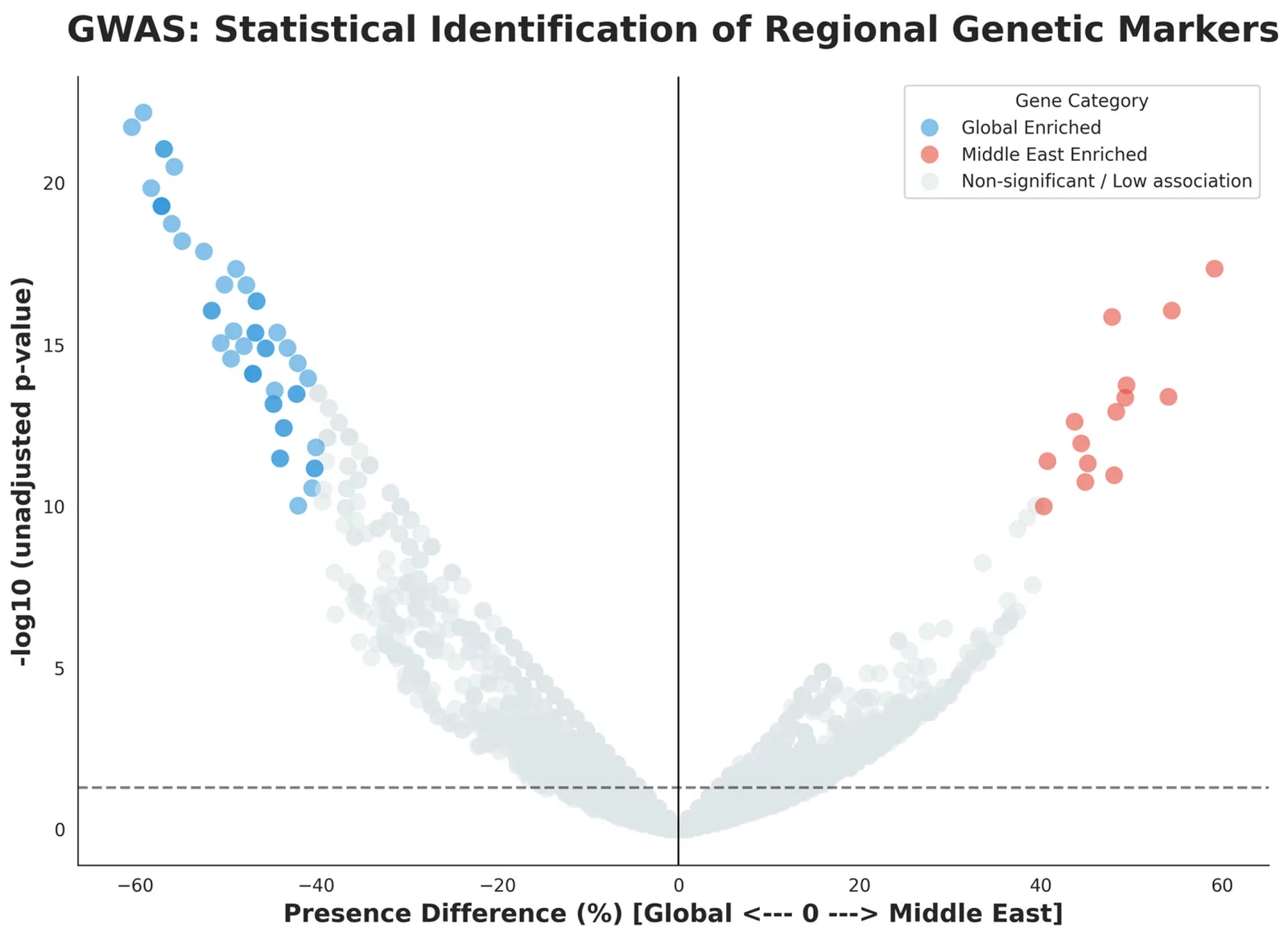
Genome-wide association study (GWAS) identifying regional genetic markers associated with Middle Eastern and Global *Acinetobacter baumannii* populations. The volcano plot displays the statistical association of 10,971 accessory genes with geographic origin. The x-axis represents the difference in gene prevalence (%) between cohorts, with negative values indicating enrichment in the Global cohort and positive values indicating enrichment in the Middle Eastern cohort. The y-axis represents statistical significance as −log10 (unadjusted *p*-value). Genes meeting the predefined criteria for regional association (Bonferroni-adjusted *p* < 0.05 and an absolute prevalence difference > 40%) are highlighted as Global-enriched (blue) or Middle East-enriched (red), whereas genes not meeting these criteria are shown in grey as non-significant or low-association markers. The distribution of significantly associated genes demonstrates clear regional genomic differentiation between the two populations. GWAS, genome-wide association study; %, percentage; *p*-value, probability value. Dashed line indicates the significance threshold used for identifying region-associated markers.

GWAS further quantified this divergence (Figure 6). The volcano plot highlighted a clear separation between genes enriched in Middle Eastern isolates (right side, red) and those enriched in global isolates (left side, blue). Statistical significance reached levels as high as *p* = 1 × 10^−20^ for several markers. The upper-right and upper-left quadrants contained highly significant genes associated with the Middle Eastern and global populations, respectively.

Specifically, genes such as *ant1_2~aadA*, *pitA_1*, and *thyA* were significantly associated with global isolates. In the Middle Eastern cohort, approximately 15–20 significantly enriched genes were identified, many of which showed frequency differences of 40–50% compared to global isolates. The GWAS results demonstrated a clear demarcation between region-specific markers and non-significant genes, with highly significant genomic signatures linked directly to geographic origin.

### 3.6. Machine Learning Classification of Regional Populations

To assess the ability of accessory genome variation to discriminate between geographic groups, a Random Forest classifier was trained using the accessory pangenome matrix. The model achieved a classification accuracy of 79.89% for distinguishing Middle Eastern isolates from the global cohort based on five-fold cross-validation. Feature importance analysis using Gini importance identified variants of the *ISAba1* insertion sequence and the *ampC* β-lactamase gene as the highest-ranking variables contributing to model performance. These findings indicate that accessory gene presence–absence patterns differ sufficiently between the Middle Eastern and global cohorts to enable classification based on genomic content.

The most prominent region-associated markers identified via GWAS are summarized in Table 4. Variants of the *ISAba1* insertion sequence emerged as the strongest Middle Eastern markers, appearing in 66% of Middle Eastern isolates but in less than 7% of global isolates (*p* = 4.4 × 10^−14^). Additionally, the *tufB* gene showed strong enrichment among Middle Eastern isolates, being present in 99% of Middle Eastern genomes compared with 51.1% of Global genomes. Other significantly associated genes were involved in MGE, transport systems, and metabolic pathways, further highlighting the prevalence differences between the two cohorts.

### 3.7. Temporal Dynamics of Mobile Genetic Elements

Temporal analysis of MGE density (Supplementary Figures S1 and S2) indicated that mobilome levels remained relatively stable over the study period for both populations. However, Middle Eastern isolates consistently maintained a higher mobilome density (mean 20.42) than global isolates (mean 17.28). In temporal plots, the Middle Eastern cohort exhibited a synchronized distribution with minimal fluctuation, whereas the global cohort showed greater variance and broader temporal shifts. While several global isolates appeared as hyper-mobile outliers, the Middle Eastern population remained tightly clustered around its regional average.

### 3.8. Virulence Factor Distribution and Sequence Type Diversity

**Figure 7:**
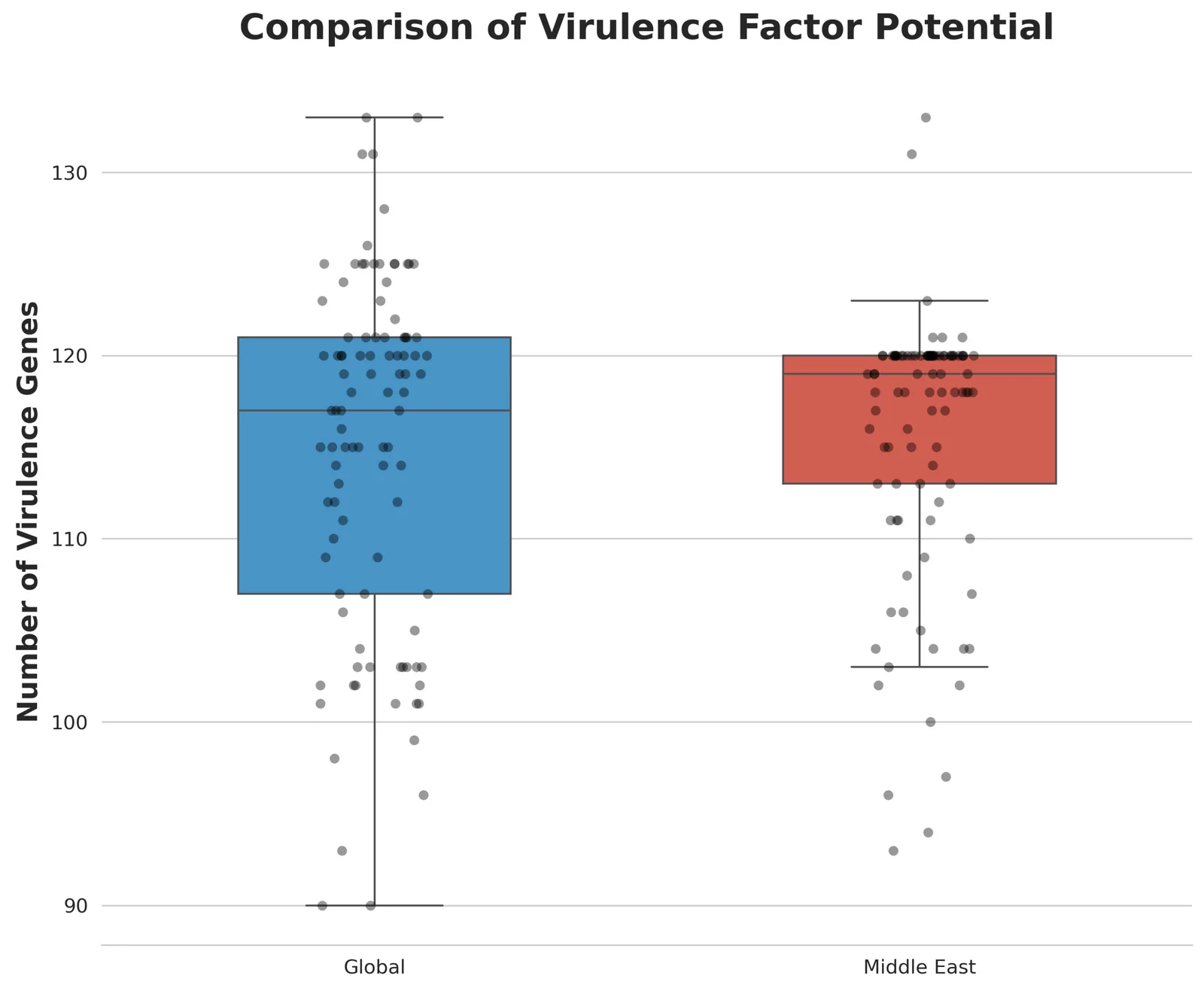
Comparison of virulence factor gene content between global and Middle Eastern *A. baumannii* isolates. Boxplot illustrating the distribution of virulence factor genes identified in global and Middle Eastern isolates using the VFDB. Each point represents an individual genome. Both populations exhibited similar median virulence gene counts, indicating comparable overall virulence potential. However, Middle Eastern isolates displayed a narrower distribution and lower variability, whereas global isolates showed a broader range of values and greater dispersion. These findings suggest that virulence gene content is more conserved within the Middle Eastern population despite comparable average virulence factor abundance between the two cohorts.

Virulence factor distribution, assessed via the VFDB, is presented in Figure 7. All isolates maintained a high baseline of virulence determinants, with a median of 119 genes. However, as shown in Table 5, the distribution width varied by region, global isolates ranged from approximately 90 to 133 genes, while Middle Eastern isolates were more concentrated between 113 and 120 genes. The variance was lower in the Middle Eastern group (SD = 7.27) compared to the global group (SD = 9.75). Despite these distributional differences, the mean virulence gene counts were similar (115.62 for Middle Eastern vs. 114.7 for global), indicating that while the average density is comparable, the Middle Eastern population exhibits greater convergence. As shown in Table 6, sequence type analysis revealed that ST2, a member of the IC2 lineage, accounted for 66% of Middle Eastern isolates and 65.7% of global isolates. This dominance of ST2 is consistent with the compact clustering observed in PCA and the conserved patterns identified in the accessory genome cluster map. Together, these findings revealed a consistent regional genomic pattern characterized by reduced genomic diversity, conserved accessory-genome organization, and enrichment of region-associated genetic markers within an ST2/IC2-dominated population.

To further examine the functional composition of the virulome, virulence-associated genes were categorized into six major functional groups (Table 7). Middle Eastern isolates showed higher mean abundances of biofilm-associated genes (12.20 vs. 11.17 genes per isolate) and secretion-system-associated genes (11.97 vs. 10.51), whereas iron-acquisition-associated genes were more abundant in global isolates (25.18 vs. 19.03). Adhesin-associated genes (3.99 vs. 4.11), capsule-associated genes (0.98 vs. 0.99), and persistence-associated genes (0.98 vs. 1.00) showed broadly similar mean abundances between the two populations. These findings indicate that comparable overall virulence-factor counts may nevertheless be accompanied by differences in the composition of specific virulence-associated categories.

The distribution of acinetobactin-associated siderophore genes identified through VFDB screening is presented in Table 8. The *bas* genes (*basA–basJ*), associated with acinetobactin biosynthesis, were highly prevalent in both cohorts. Among Middle Eastern isolates, their prevalence ranged from 90.0% (*basI*) to 100.0% (*basB*), whereas the corresponding prevalence in Global isolates ranged from 97.0% (*basI*) to 100.0% for several genes. The *bau* genes associated with acinetobactin uptake were also widely distributed. *bauA* was detected in 79.0% of Middle Eastern isolates and 71.0% of Global isolates, while *bauB–bauF* were detected in 97.0–99.0% of Middle Eastern isolates and 100.0% (*bauB–bauF*) of Global isolates, except for the slightly lower Middle Eastern frequencies. Overall, the acinetobactin-associated genes showed high prevalence across both cohorts, with most individual genes detected in more than 90% of isolates.

### 3.9. Temporal Dynamics of Antimicrobial Resistance Gene Burden

Supplementary Figure S3 illustrates the distribution of AMR gene counts among Middle Eastern *A. baumannii* isolates collected between 2008 and 2016. Resistance gene counts ranged from approximately 15 to 47 genes per genome, with most isolates carrying between 25 and 35 resistance determinants. Similar resistance gene burdens were observed across all sampling years, and substantial overlap was evident between yearly distributions.

The highest resistance gene count was detected in a 2013 isolate, which carried approximately 47 resistance genes, whereas the lowest count was observed in a 2016 isolate with approximately 15 genes. Isolates collected in 2013 and 2016 displayed the widest range of resistance gene counts, while isolates from 2015 were more tightly clustered around 30–35 genes per genome (Supplementary Figure S3).

### 3.10. Distribution of Antimicrobial Resistance Determinants

**Figure 8:**
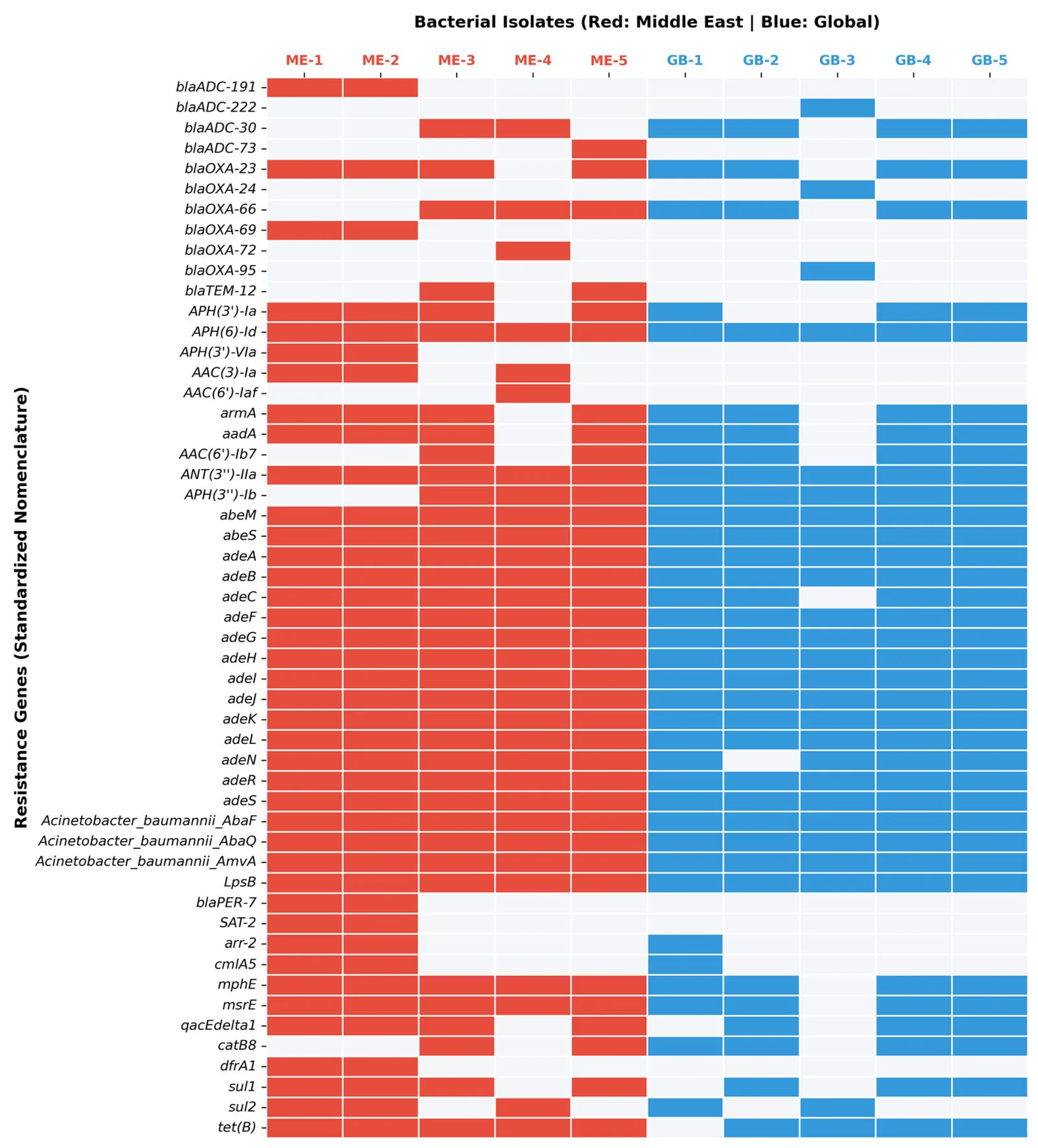
Distribution of AMR determinants among representative Middle Eastern and Global *A. baumannii* isolates. The figure highlights the distribution of carbapenem-associated β-lactamase determinants, including OXA- and ADC-type genes, together with aminoglycoside resistance genes, efflux-associated determinants, and resistance markers associated with macrolides, sulfonamides, tetracyclines, and other antimicrobial classes. *bla*OXA-23 was detected in representatives of both cohorts, while other OXA- and ADC-type determinants showed variable distributions between the Middle Eastern and Global isolates. Additional regional differences were observed among aminoglycoside-modifying enzymes and other AMR determinants. ME, Middle East; GB, global; blaADC, *Acinetobacter* derived cephalosporinase gene; blaOXA, oxacillinase beta-lactamase gene; blaTEM, TEM family beta-lactamase gene; APH, aminoglycoside phosphotransferase gene; AAC, aminoglycoside acetyltransferase gene; armA, 16S rRNA methyltransferase gene; aadA, aminoglycoside nucleotidyltransferase gene; ANT, aminoglycoside nucleotidyltransferase gene; abeM, multidrug efflux pump gene; abeS, multidrug efflux pump gene; adeA–S, resistance-nodulation-cell division (RND) family efflux pump system genes; AbaF, efflux pump gene; AbaQ, efflux pump gene; AmvA, major facilitator superfamily efflux pump gene; LpsB, lipopolysaccharide biosynthesis protein B gene; blaPER, Pseudomonas extended-spectrum beta-lactamase gene; SAT-2, streptothricin acetyltransferase gene; arr-2, ADP-ribosyltransferase gene; cmlA5, chloramphenicol efflux pump gene; mphE, macrolide 2′-phosphotransferase E gene; msrE, ABC-F type ribosomal protection protein gene; qacEdelta1, quaternary ammonium compound resistance gene delta 1; catB8, chloramphenicol acetyltransferase gene; dfrA1, dihydrofolate reductase gene; sul1, sulfonamide resistance gene 1; sul2, sulfonamide resistance gene 2; tet(B), tetracycline resistance protein gene B.

The distribution of AMR determinants among representative Middle Eastern and Global *A. baumannii* isolates is shown in Figure 8. Carbapenem-associated β-lactamase determinants showed variable distributions between the two cohorts. *bla*OXA-23 was detected in four of the five Middle Eastern representatives and four of the five Global representatives. Other OXA-type determinants showed distinct distributions, with *bla*OXA-24 and *bla*OXA-95 detected among Global representatives, whereas *bla*OXA-69 and *bla*OXA-72 were detected among Middle Eastern representatives. *bla*OXA-66 was detected in representatives from both cohorts. Among the ADC-type β-lactamases, *bla*ADC-191 and *bla*ADC-73 were detected among Middle Eastern representatives, whereas *bla*ADC-222 was detected among a Global representative. *bla*ADC-30 was observed in representatives from both cohorts. Aminoglycoside resistance determinants also showed variable distributions across the representative isolates. *ANT(3″)-IIa* and *APH(6)-Id* were detected across representatives from both cohorts, whereas *AAC(3)-Ia*, *APH(3′)-Ia*, *aadA*, *AAC(6′)-Iaf*, *AAC(6′)-Ib7*, and *armA* showed differences in their distribution. Efflux-associated determinants, including *adeA*, *adeB*, *adeC*, *adeF*, *adeG*, *adeH*, *adeI*, *adeJ*, *adeK*, *adeL*, *adeN*, *adeR*, and *adeS*, were broadly represented across the examined isolates.

Additional resistance determinants included *PER-7*, *SAT-2*, *arr-2*, *cmlA5*, *mphE*, *msrE*, *qacEdelta1*, *catB8*, *dfrA1*, *sul1*, *sul2*, and *tet(B)*, with several showing differences in distribution between the Middle Eastern and Global representatives. *PER-7*, *SAT-2*, *arr-2*, *cmlA5*, and *dfrA1* were predominantly observed among Middle Eastern representatives, whereas *catB8* showed representation in both cohorts with several Global isolates. The distribution of *sul1*, *sul2*, and *tet(B)* also varied among the representative isolates. Overall, the representative profiles demonstrated variation in carbapenem-associated β-lactamase determinants, particularly among OXA- and ADC-type genes, together with differences in the distribution of additional AMR determinants between the Middle Eastern and Global cohorts.

## 4. Discussion

The comparative genomic analysis presented here revealed marked differences between Middle Eastern *A. baumannii* isolates and the broader global population, primarily characterized by the predominance of a stable and highly adapted ST2/IC2 lineage within the region. In contrast to the substantial genomic heterogeneity observed among globally distributed isolates, Middle Eastern isolates exhibited a comparatively conserved genomic architecture with limited variation in accessory genome composition, resistance determinants, and virulence gene profiles. Collectively, these findings suggest that the regional *A. baumannii* population has reached a relatively stable genomic state, in which adaptation appears to be driven less by the continual acquisition of novel AMR genes and more by the optimization of existing resistance mechanisms through regulatory elements such as *ISAba1*, together with the maintenance of a highly dynamic mobilome capable of facilitating ongoing genomic plasticity.

### 4.1. Genomic Conservation and Clonal Expansion Within the Middle Eastern ST2/IC2 Population

MLST analysis revealed that ST2 is the dominant lineage in both the Middle Eastern and Global cohorts, accounting for 66.0% and 65.7% of isolates, respectively. These findings demonstrate the worldwide success of the IC2/ST2 lineage. However, despite the similar prevalence of ST2 in both cohorts, Middle Eastern isolates exhibited reduced genomic diversity, more conserved accessory-genome profiles, and distinct region-associated genetic markers. These findings suggest that the distinguishing feature of the Middle Eastern population is not ST2 prevalence itself, but the presence of a more genomically conserved ST2-associated population characterized by unique accessory-genome composition and regional genomic signatures. This suggests that, despite the shared predominance of ST2, the Middle Eastern population exhibits distinct regional genomic characteristics relative to the global cohort, resulting in a synchronized genomic signature that distinguishes it from global ST2 counterparts. The regional dominance of the ST2/IC2 lineage is consistent with previous reports from the Middle East. Brek et al. reported that 81.2% of carbapenemase-producing *A. baumannii* isolates from Saudi Arabia belonged to ST2 [35], while Darwiche et al. found that 91.5% of isolates recovered during a tertiary-care hospital outbreak in Lebanon were ST2 within IC2 [36]. These findings support the importance of ST2/IC2 as a major lineage in the regional *A. baumannii* population.

The markedly lower genomic diversity observed among Middle Eastern isolates compared with the global cohort may partly reflect the expansion and persistence of this successful lineage. Li et al. described ST2 as a major component of an epidemic super-lineage responsible for a substantial proportion of global clinical *A. baumannii* isolates, highlighting its epidemiological success [11]. In the present study, the combination of ST2 predominance and reduced genomic variation suggests that a relatively conserved ST2/IC2 lineage has become established within the sampled Middle Eastern population. The close genetic relatedness of isolates from different countries may be compatible with regional dissemination of related ST2/IC2 lineages or persistence of a shared ancestral population. However, genomic similarity alone cannot establish specific intra-hospital, inter-hospital, or cross-border transmission events, which require temporally resolved genomic sampling and epidemiological information. Thus, the observed genomic structure should be interpreted as evidence of a conserved regional population structure rather than direct evidence of transmission.

The predominance and genomic conservation of ST2/IC2 nevertheless have important infection-prevention and control implications. The observed pattern suggests that the persistence and dissemination of an established high-risk lineage may be an important consideration when interpreting the regional genomic structure, rather than assuming that regional resistance patterns necessarily result from continuous acquisition of new resistance determinants. Accordingly, clonal dissemination should be considered an important target for infection-control strategies, particularly within and between healthcare facilities. Genomic surveillance could also be useful for monitoring the persistence and geographic distribution of dominant ST2/IC2 lineages over time and for distinguishing clonal dissemination from the emergence of newly acquired resistance determinants. Integration of genomic epidemiology with antimicrobial stewardship may further help contextualize changes in resistance and determine whether they are associated primarily with clonal expansion or acquisition of additional resistance determinants.

In this study antimicrobial consumption and infection-prevention practices were not assessed. The reduced diversity observed in the Middle Eastern cohort may reflect a combination of ST2/IC2 expansion, regional dissemination, and shared selective pressures. These findings are consistent with the population-bottleneck hypothesis proposed by Karah et al., whereby successful epidemic clones may become dominant during regional dissemination and subsequently maintain relatively stable genomic characteristics [37]. The use of publicly available NCBI genome assemblies may introduce some sampling bias, as clinically important or outbreak-associated isolates may be more frequently represented in genomic databases. This factor may have influenced the observed genomic diversity within the Middle Eastern cohort. Therefore, the findings should be interpreted within the context of the available dataset. These findings indicate that the regional genomic structure identified in this study has both biological and epidemiological significance, while the machine-learning analysis provides complementary evidence for the discriminatory value of these genomic features.

### 4.2. Stable Accessory Genome Despite a Large Accessory Pangenome

A notable finding of this study was the remarkable stability of the Middle Eastern accessory genome despite *A. baumannii* possessing a highly expansive accessory pangenome. Our pangenome analysis identified 10,971 non-redundant genes, with the cloud genome (genes present in <15% of isolates) accounting for 61.0% of the total genetic repertoire. This extensive accessory gene pool is characteristic of an “open” pangenome and reflects the considerable genomic plasticity of *A. baumannii*, consistent with the observations of Pearl et al., However, despite this species-wide genomic diversity, Middle Eastern isolates consistently clustered into three major groups characterized by highly conserved accessory gene profiles, indicating substantially lower genomic variability than that observed among globally distributed isolates [9].

These findings suggest that although *A. baumannii* as a species retains a considerable capacity for gene acquisition and loss, the dominant ST2/IC2 lineage circulating in the Middle East has maintained a relatively conserved accessory genome that is likely associated with successful adaptation to the regional healthcare environment. A similar pattern of increasing genomic stability has been reported by Gopikrishnan et al., who observed evidence of “genomic streamlining” in contemporary *A. baumannii* populations, characterized by reduced overall gene content together with increased conservation of the core genome. Although their study examined temporal rather than geographic evolution, both studies support the concept that successful epidemic populations can retain an open pangenome while simultaneously exhibiting increasing genomic stability [38]. Our findings therefore suggest that the remarkable diversity of the *A. baumannii* pangenome is primarily maintained by less common lineages, whereas the dominant Middle Eastern ST2 population has retained a highly conserved accessory gene repertoire that may contribute to its long-term persistence and epidemiological success.

### 4.3. ISAba1 Enrichment and Regulatory Optimization

A notable feature of the Middle Eastern *A. baumannii* population was the significant enrichment of the insertion sequence ISAba1, which emerged as the strongest region-associated marker in the GWAS. The significant enrichment of ISAba1 (*p* < 10^−14^) in the Middle Eastern cohort, despite the similar predominance of ST2 in both cohorts, is a major finding. This finding suggests that the Middle Eastern ST2-dominated population has experienced greater enrichment of insertion sequences. While Global ST2 isolates may carrysimilar resistance genes, the higher prevalence of ISAba1 in the Middle Eastern cohort may contribute to enhanced expression of adjacent resistance determinants, including carbapenemases such as blaOXA-23. ISAba1 was detected in approximately 66% of Middle Eastern isolates but in fewer than 7% of globally distributed isolates, highlighting a marked regional difference in its prevalence. This enrichment is biologically relevant because ISAba1 can function not only as a mobile genetic element but also as a regulatory sequence. As described by Pearl et al. and Müller et al., ISAba1 can provide a strong outward-facing promoter when inserted upstream of resistance genes such as *blaOXA-23* and *blaADC*, potentially enhancing expression of these β-lactamases and contributing to carbapenem resistance [9,39].

The resistance-gene profiles further highlight the potential importance of carbapenem-associated β-lactamases in shaping the resistance characteristics of the regional population. The detection of *blaOXA-23* in representatives from both cohorts may reflect the widespread distribution of this carbapenemase among *A. baumannii* populations, whereas the occurrence of *blaOXA-24* and *blaOXA-72* primarily among Global representatives may indicate differences in the distribution of OXA-type carbapenemases between the sampled populations. In the Middle Eastern representatives, the presence of *blaOXA-69*, an *OXA-51*-like intrinsic β-lactamase variant, together with additional determinants such as *blaADC-191* and *blaPER-7*, may contribute to the distinct resistance-gene profile observed in this cohort. Similarly, the detection of *blaADC-222* among Global representatives may reflect differences in the accessory resistance repertoire between the two populations. Overall, these patterns suggest that although carbapenem resistance-associated determinants are shared across the cohorts, the specific composition of β-lactamase determinants may vary between populations. The prominence of *blaOXA-23* further supports the potential importance of established carbapenemase-associated resistance mechanisms in the resistance repertoire of *A. baumannii*, rather than indicating that resistance differences are necessarily driven by a single β-lactamase determinant [16,40].

Our findings suggest that Middle Eastern isolates may rely more heavily on the maintenance and potential optimization of existing resistance mechanisms than on the accumulation of additional resistance genes. This interpretation is supported by the observation that Middle Eastern isolates contained fewer AMR genes on average (30.09 genes per genome) than globally distributed isolates (37.63 genes per genome). The enrichment of *ISAba1* may contribute to the regulation of key resistance determinants, although functional expression was not assessed in the present study. These findings are consistent with a model in which regulatory variation in existing resistance mechanisms may contribute to the persistence and success of the regional ST2/IC2 lineage [39,41].

### 4.4. The Inverse Relationship: Fewer Resistance Genes, Higher Mobilome

Despite carrying fewer AMR genes, Middle Eastern *A. baumannii* isolates exhibited a higher abundance of mobility-associated genes (mean 20.42) than globally distributed isolates (mean 17.28). This apparent inverse relationship is unlikely to indicate that the regional population has a lower capacity for genetic adaptation. Rather, it may indicate that the dominant Middle Eastern ST2/IC2 population has maintained genomic plasticity through MGE while relying on an already established resistance repertoire. In this context, a larger mobilome does not necessarily imply a greater requirement for continuous acquisition of AMR genes; mobile elements may facilitate genome remodeling, gene rearrangement, and the acquisition or maintenance of accessory functions that provide advantages under changing environmental or clinical conditions [42,43]. Functional annotation of the 1102 Middle East-specific genes further demonstrated the prominence of mobility-associated functions, with HGT-associated genes and MGEs, including transposases, integrases, and insertion sequences, representing the largest characterized functional category. Collectively, these findings suggest that mobility-associated genetic features may contribute to the genomic plasticity and differentiation of the regional accessory genome, although their specific contributions to long-term adaptation cannot be established from the present analysis alone [44].

The apparent combination of higher mobility-associated content and lower AMR-gene burden may therefore reflect functions of MGE that extend beyond AMR [9]. Such elements may be maintained because they provide adaptive functions beyond resistance, including genomic remodeling and the acquisition or maintenance of accessory traits that may support persistence under changing clinical or environmental conditions. The enrichment of *ISAba1* provides a more specific possible mechanism for maintaining resistance without requiring expansion of the AMR repertoire. Because *ISAba1* can provide promoter activity when positioned upstream of certain resistance genes, its higher prevalence in the Middle Eastern cohort could potentially alter the expression of existing resistance determinants [45]. Under this model, evolutionary advantage may arise not from acquiring more resistance genes, but from modifying the regulation or genomic context of resistance determinants that are already present [46]. However, because gene expression and promoter activity were not experimentally assessed in this study, this interpretation remains hypothetical [47]. A complementary explanation is that the dominant ST2/IC2 lineage may represent an established successful population whose resistance repertoire has already provided substantial adaptive benefit. Once a lineage has acquired an effective combination of resistance determinants, further acquisition of additional genes may provide diminishing selective returns while increasing the genetic and metabolic burden associated with maintaining unnecessary determinants. Maintaining mobile genetic capacity, in contrast, could preserve the ability to respond to future selective pressures without requiring constitutive expansion of the resistance repertoire. This model would explain why mobility-associated content and AMR-gene burden do not necessarily increase in parallel and is compatible with the observed combination of ST2/IC2 predominance, reduced genomic diversity, and lower AMR-gene abundance in the Middle Eastern cohort [48]. Under this scenario, acquisition of additional resistance determinants may provide limited incremental benefit while potentially imposing fitness costs, whereas maintaining genomic plasticity may remain advantageous under changing selective pressures.

Our findings are consistent with previous reports highlighting the importance of HGT in the evolution of *A. baumannii*. Borgio described an XDR Saudi Arabian isolate containing plasmid sequences with high similarity to those identified in other clinically important pathogens, including *Klebsiella pneumoniae*, supporting the occurrence of interspecies HGT [49]. Rather than functioning solely as vehicles for the acquisition of AMR genes, the abundance of MGE observed in our study suggests that they also contribute to the continuous reshaping of the accessory genome. This interpretation is reinforced by the predominance of HGT-associated genes among Middle East-specific accessory genes, indicating that regional genomic differentiation has been driven not only by the dissemination of resistance determinants but also by the acquisition, maintenance, and rearrangement of accessory genetic material [50]. The relatively narrow distribution of mobility-associated feature counts among Middle Eastern isolates may further suggest that this characteristic is shared across the dominant regional ST2/IC2 population rather than being driven solely by a small number of genomes [51]. Importantly, the mobility-associated counts reported here were derived from genome annotations rather than from a dedicated comprehensive mobilome-characterization pipeline. Therefore, they should be interpreted as mobility-associated genomic content rather than a direct measure of the complete mobilome or its activity. In particular, the presence of genes associated with mobility does not establish that the corresponding elements are intact, actively mobile, or undergoing transposition. Nevertheless, the consistent difference between the two cohorts provides evidence for a higher abundance of mobility-associated genetic features in the sampled Middle Eastern population.

### 4.5. Genomic Equilibrium and Evolutionary Stability

Our comparative genomic analysis revealed a remarkably consistent pattern of resistance gene abundance among Middle Eastern isolates collected between 2008 and 2016. Rather than demonstrating substantial differences in the overall resistance-gene repertoire, the Middle Eastern isolates showed a relatively conserved AMR profile, which may reflect relative genomic stability within the dominant regional lineage. This pattern is consistent with observations reported for successful epidemic clones, which can achieve long-term epidemiological persistence after acquiring genomic features that are well suited to their ecological and clinical environments [9,11,12].

Collectively, these findings suggest that the established resistance repertoire may be sufficient to maintain the genomic configuration of the dominant regional lineage without requiring extensive additional acquisition of resistance determinants

### 4.6. Limited Evidence for a Fitness Trade-Off

One of the clinically relevant observations of this study was the absence of a clear reduction in virulence-associated gene content among the Middle Eastern *A. baumannii* population despite its extensive AMR burden [52]. Traditionally, the acquisition of extensive AMR has been proposed to reduce bacterial fitness or virulence because of the metabolic costs associated with maintaining multiple resistance determinants. However, despite exhibiting extensive AMR, Middle Eastern isolates retained a consistently high repertoire of virulence-associated genes (median = 119 genes) together with a high abundance of mobility-associated genetic features, suggesting that resistance-associated genomic adaptations have not been accompanied by a marked reduction in virulence gene content [38].

In addition to maintaining a high number of virulence-associated genes, Middle Eastern isolates displayed substantially lower variability in virulence gene profiles than globally distributed isolates, indicating a highly conserved virulome within the dominant ST2/IC2 lineage. A similar relationship between AMR and virulence has been reported by Aranzamendi et al., who demonstrated that the persistence and epidemiological success of IC2 clones are associated with the combined maintenance of resistance determinants and virulence factors, including the KL32 capsule, which contributes to bacterial survival [53]. Although our study did not evaluate virulence phenotypes experimentally, the simultaneous conservation of resistance determinants, virulence-associated genes, and mobility-associated genetic features suggests that the dominant Middle Eastern ST2 lineage has maintained multiple genomic traits without clear evidence of a reduction in overall virulence-associated gene content [54]. Although the two cohorts had comparable overall numbers of virulence-associated genes, category-level analysis revealed differences in their functional composition. Middle Eastern isolates showed higher mean abundances of biofilm- and secretion-system-associated genes, whereas Global isolates showed higher mean abundance of iron-acquisition-associated genes. In contrast, adhesin-, capsule-, and persistence-associated gene abundances were broadly similar. These findings indicate differences in the representation of specific virulence-associated functions rather than differences in the overall size of the virulence repertoire.

These category-level differences may have potential implications for the ecological and clinical behavior of the two populations. The higher abundance of biofilm-associated genes in Middle Eastern isolates may indicate greater genomic potential for persistence on biotic and abiotic surfaces, which could contribute to prolonged colonization and survival in healthcare environments [55]. Similarly, the higher representation of secretion-system-associated genes may reflect differences in the genomic potential for host–pathogen interactions and invasive processes [56]. The broadly similar abundances of adhesin-, capsule-, and persistence-associated genes suggest that these functions are relatively conserved and are unlikely to explain the major differences between the cohorts at the level of gene content alone. However, gene presence or abundance does not establish gene expression, functional activity, colonization capacity, invasive potential, environmental persistence, or transmission efficiency. Functional and phenotypic studies would therefore be required to determine whether the observed genomic differences translate into clinically meaningful differences in pathogenicity or transmission [4]. Conversely, iron-acquisition-associated genes were more abundant in the Global cohort. Among these determinants, siderophore-associated genes involved in the acinetobactin system, which mediates high-affinity iron acquisition under iron-limited host conditions, were highly prevalent across both cohorts, including the *basA–basJ* genes associated with acinetobactin biosynthesis and the *bauA–bauF* genes associated with acinetobactin uptake [57], the high prevalence of these genes in both populations indicates that acinetobactin-associated iron acquisition represents a conserved component of the *A. baumannii* virulence repertoire in the analyzed dataset [58]. However, the higher cumulative abundance of auxiliary iron-acquisition genes in the Global cohort may reflect a broader representation of iron-uptake functions in these isolates, although the biological significance of this difference remains uncertain. Importantly, gene presence or abundance alone does not establish functional expression or phenotypic activity, and therefore the observed difference should not be interpreted as evidence of greater iron-acquisition capacity or virulence in the Global isolates.

These results identify population-level differences in the distribution of virulence-associated genomic features rather than direct differences in pathogenicity, colonization, persistence, or transmission. The observed patterns should therefore be considered hypothesis-generating. Functional validation through transcriptomic or proteomic analyses and phenotypic assays, including biofilm, adhesion, secretion, and iron-limitation studies, would be required to determine whether these genomic differences translate into clinically relevant phenotypes.

### 4.7. Region-Specific Genomic Fingerprint and Surveillance Potential

Our GWAS and pangenome analyses identified a distinct genomic signature that differentiates Middle Eastern *A. baumannii* isolates from globally distributed populations. This regional genomic fingerprint comprises 1102 Middle East-specific genes, significant enrichment of *ISAba1*, and the near-ubiquitous presence of the *tufB* gene (99% prevalence). Importantly, this regional genomic fingerprint is not explained solely by the predominance of ST2, as ST2 was similarly prevalent in the Global cohort. Rather, the distinguishing characteristics arose from differences in accessory-genome composition, ISAba1 enrichment, and the reduced genomic heterogeneity observed within the Middle Eastern ST2 population.

The high prevalence of *tufB* together with the marked enrichment of *ISAba1* suggests that these genetic markers may have potential utility in future molecular surveillance strategies. Unlike the greater genomic heterogeneity observed among globally distributed isolates, the relative homogeneity of the Middle Eastern population may facilitate the development of region-specific molecular assays capable of rapidly identifying the dominant ST2/IC2 lineage in clinical samples. Such approaches could improve outbreak investigations and assist in monitoring inter-hospital transmission, which Duan et al., identified as an important contributor to the dissemination of nosocomial *A. baumannii* [12]. Furthermore, the identification of 1102 region-specific accessory genes raises the possibility that genomic signatures could eventually be used for geographic source attribution of clinical isolates, thereby strengthening international surveillance efforts in an era of increasing global travel and cross-border dissemination of MDR pathogens, as discussed by Müller et al. [39].

### 4.8. Uncharacterized Accessory Genes: Functional Insights and Future Directions

A prominent feature of the Middle East-specific accessory genome was the high proportion of initially uncharacterized genetic material. As illustrated in Figure 3, 978 of the 1102 regional genes (88.9%) lacked functional annotation during the primary analysis. Following protein-sequence validation, 915 high-quality sequences were retained for secondary characterization, with recognizable conserved-domain signatures identified in 424 (46.3%) proteins. Most resolved proteins were associated with metabolic or enzymatic functions, while additional domains were associated with prophage-related functions, mobile DNA and plasmid stability, persistence and toxin–antitoxin systems, stress response and DNA repair, and transcriptional regulation.

The regional-specific nature of these genes may reflect recently acquired accessory elements, lineage-specific functions, or mobile-element-associated sequences, although their acquisition history cannot be established from the present analysis alone. The identification of domains associated with toxin–antitoxin systems and SOS-response regulation, including *Lex*A-associated domains, provides additional functional context for the previously uncharacterized regional accessory repertoire [59]. Some of these functions could potentially contribute to persistence or adaptation in healthcare-associated environments; however, their contribution to hospital adaptation requires experimental validation. These findings suggest that a subset of the proteins initially classified as hypothetical has recognizable functional or evolutionary associations that were not captured by the primary annotation. However, conserved-domain assignments represent putative functional associations and do not establish a specific phenotype or mechanism of regional adaptation. The remaining 491 validated proteins lacked recognizable conserved-domain assignments and therefore remain functionally unresolved. These proteins may include poorly characterized lineage-specific proteins, mobile-element-associated proteins, or other determinants that are currently not represented in the databases used. Further characterization using complementary computational, transcriptomic, structural, and experimental approaches will be necessary to determine their biological roles.

### 4.9. Greater Genomic Heterogeneity Among Global Isolates

The relative genomic homogeneity of the Middle Eastern *A. baumannii* population contrasted markedly with the substantially greater heterogeneity observed among globally distributed isolates. Compared with the Middle Eastern cohort, global isolates exhibited greater variation in resistance gene content, mobilome composition, and accessory genome profiles, reflecting a more diverse genomic landscape. This increased diversity was also evident in the hierarchical clustering heatmap, where global isolates displayed fragmented and less cohesive patterns of accessory gene distribution, in contrast to the dense clusters observed among Middle Eastern ST2 isolates.

The greater genomic diversity observed in the global population likely reflects the influence of distinct evolutionary histories and selective pressures operating across different geographical regions. Müller et al., similarly reported considerable regional variation in the distribution of International Clones and carbapenemase genes, with certain lineages predominating in specific parts of the world, such as the increased prevalence of IC5 in Latin America [39]. Likewise, Pagano et al., described *A. baumannii* as possessing a highly mobile and “promiscuous” resistome characterized by frequent HGT and continuous exchange of accessory genetic material among diverse lineages [46]. Consistent with these observations, the global isolates included in our study appear to represent populations undergoing ongoing genomic diversification, whereas the Middle Eastern population was characterized by a comparatively conserved ST2/IC2 genomic background with substantially lower variability. These findings further support the conclusion that regional *A. baumannii* populations may follow distinct evolutionary trajectories shaped by local epidemiological and ecological conditions.

### 4.10. Clinical and Epidemiological Implications

The predominance and genomic conservation of the ST2/IC2 lineage within the Middle Eastern *A. baumannii* population highlight the importance of region-specific genomic surveillance. Continuous monitoring of lineage composition, resistance determinants, and accessory-genome evolution could help identify the persistence and dissemination of successful high-risk clones, consistent with the recommendations of Li et al. regarding surveillance of epidemic *A. baumannii* lineages [11].

The marked enrichment of *ISAba1* among Middle Eastern isolates also has potential implications for molecular diagnostics and surveillance. Because *ISAba1* can enhance the expression of carbapenem resistance genes depending on its genomic location, screening strategies that consider both the presence of resistance genes and their surrounding genetic context may provide more informative assessments of resistance potential than detection of resistance genes alone. Furthermore, the conserved repertoire of virulence-associated genes observed among Middle Eastern ST2 isolates suggests that these strains should be regarded as high-risk clinical lineages requiring continued surveillance and rigorous infection control measures. Although functional virulence was not assessed in the present study, the combination of extensive AMR, a highly conserved virulome, and a higher abundance of genes associated with genome mobility underscores the clinical importance of sustained genomic monitoring of this dominant regional lineage. The Random Forest model achieved a classification accuracy of 79.89%, providing complementary evidence that the identified genomic features can distinguish the Middle Eastern and Global cohorts. These region-associated genomic signatures may therefore have potential utility in genomic surveillance and outbreak investigations, although their application for geographic source attribution would require validation using larger and independently sampled datasets.

## 5. Conclusions

In conclusion, our study demonstrates that the Middle Eastern *A. baumannii* population is characterized by a distinct regional genomic signature within a globally prevalent ST2/IC2 lineage. Although ST2 was predominant in both the Middle Eastern and Global cohorts, the Middle Eastern population exhibited reduced genomic diversity, a distinct accessory-genome profile, and significant enrichment of the *ISAba1* insertion sequence. Therefore, the principal distinguishing feature of the Middle Eastern population was not ST2 prevalence itself, but its reduced genomic diversity, conserved accessory-genome structure, enrichment of *ISAba1*-associated markers, and the presence of region-specific accessory genes. This regional genomic signature was further characterized by differences in antimicrobial-resistance determinants, mobility-associated features, and virulence-associated genes. The Middle Eastern isolates maintained a relatively conserved genomic repertoire, including a high abundance of mobility-associated features and a conserved set of virulence-associated determinants, while carrying fewer AMR genes overall than the Global cohort. The enrichment of *ISAba1* represents an important component of the regional genomic signature and may contribute to the regulation of adjacent resistance determinants, although functional expression was not assessed in the present study. Similarly, the observed conservation of virulence-associated genes does not establish the absence of a phenotypic fitness trade-off. Overall, these findings indicate that the Middle Eastern *A. baumannii* population possesses a distinctive and relatively conserved genomic configuration within an ST2/IC2-dominated population. Continued genomic surveillance, together with functional and epidemiological studies, will be important for determining the biological and clinical significance of these regional genomic characteristics. Future investigations should also characterize the substantial reservoir of hypothetical proteins identified within the regional genomic signature to determine their potential functional relevance.

## Figures and Tables

**Table 1:** Pangenome composition of the 199 *A. baumannii* genomes analyzed in this study.

Pangenome Category	Number of Genes
Core genes	1495
Soft-core genes	964
Shell genes	1825
Cloud genes	6687
Total genes	10,971

**Table 2:** Comparative resistance gene burden and mobilome content among Middle Eastern and global *A. baumannii* isolates.

Group	Number of Isolates (*n*)	Resistance Gene Count (Mean ± SD)	Mobile Elements (Mean ± SD)
Global	99	37.63 ± 9.67	17.28 ± 20.72
Middle East	100	30.09 ± 4.53	20.42 ± 17.87

**Table 3:** Secondary functional characterization of Middle East-specific proteins initially.

Functional Category	No. of Proteins	Percentage of Resolved Proteins (%)
Other resolved functions (metabolic/enzymatic)	358	84.4
Prophage-associated	29	6.8
Mobile DNA and plasmid stability	16	3.8
Persistence and toxin–antitoxin	8	1.9
Stress response and DNA repair	8	1.9
Transcriptional regulation	5	1.2
Total resolved	424	100.0

**Table 4:** Most significant genomic markers identified by GWAS analysis.

Gene	Annotation	Middle East (%)	Global (%)	Bonferroni-Adjusted *p*-Value	OR
*group_1310*	IS4 family transposase *ISAba1*; hypothetical protein	66.0	6.8	4.40 × 10^−14^	26.53
*group_2522*	IS4 family transposase *ISAba1*; hypothetical protein	59.0	4.5	8.69 × 10^−13^	30.22
*tufB_2—tufA*	Elongation factor Tu 2	99.0	51.1	1.37 × 10^−12^	94.60
*group_2505*	IS4 family transposase *ISAba1*; hypothetical protein	54.0	4.5	1.77 × 10^−10^	24.65
*group_835*	hypothetical protein	70.0	15.9	4.03 × 10^−10^	12.33
*group_2485*	IS4 family transposase *ISAba1*; hypothetical protein	55.0	5.7	7.86 × 10^−10^	20.14
*group_2441*	IS4 family transposase *ISAba1*; hypothetical protein	54.0	5.7	1.17 × 10^−9^	19.47
*group_2481*	IS4 family transposase *ISAba1*; hypothetical protein	46.0	2.3	2.37 × 10^−9^	36.63
*group_1633*	IS4 family transposase *ISAba1*; hypothetical protein	49.0	4.5	1.17 × 10^−8^	20.98
*group_2469*	IS4 family transposase *ISAba1*; hypothetical protein	43.0	2.3	3.94 × 10^−8^	32.44

Abbreviations: *p*-value, probability value; OR, odds ratio; IS4, insertion sequence 4; ISAba1, insertion sequence *Acinetobacter baumannii* 1; tufB_2, elongation factor Tu gene B variant 2; tufA, elongation factor Tu gene A.

**Table 5:** Comparison of virulence factor gene distribution between Middle Eastern and global *A. baumannii* isolates.

Group	Mean Virulence Gene Count	SD	Minimum	Maximum
**Global**	114.79	9.75	90	133
**Middle East**	115.62	7.27	93	133

**Table 6:** Distribution of the most prevalent sequence types among Middle Eastern and Global *A. baumannii* isolates.

Sequence Type	Middle East *n*	Middle East %	Global *n*	Global %
**ST2**	66	66.0	65	65.7
**ST1580**	6	6.0	—	—
**Unassigned (–)**	6	6.0	4	4.0
**ST636**	4	4.0	—	—
**ST1**	3	3.0	—	—
**ST499**	—	—	11	11.1

Abbreviations: ST, sequence type; *n*, sample size.

**Table 7:** Functional categorization of virulence-associated genes in Middle Eastern and global *A. baumannii* isolates.

Virulence Category	Middle East (Mean Genes/Isolate)	Global (Mean Genes/Isolate)
Adhesins	3.99	4.11
Biofilm Formation	12.20	11.17
Capsule Synthesis	0.98	0.99
Iron Acquisition	19.03	25.18
Persistence Mechanisms	0.98	1.00
Secretion Systems	11.97	10.51
Other Virulence	67.46	71.54

**Table 8:** Prevalence of acinetobactin-associated siderophore genes among Middle Eastern and Global *A. baumannii* isolates.

Gene	Function/Association	Global (%)	Middle East (%)
*basA*	Acinetobactin biosynthesis	99.0	97.0
*basB*	Acinetobactin biosynthesis	100.0	100.0
*basC*	Acinetobactin biosynthesis	99.0	97.0
*basD*	Acinetobactin biosynthesis	99.0	94.0
*basF*	Acinetobactin biosynthesis	100.0	98.0
*basG*	Acinetobactin biosynthesis	100.0	97.0
*basH*	Acinetobactin biosynthesis	100.0	97.0
*basI*	Acinetobactin biosynthesis	97.0	90.0
*basJ*	Acinetobactin biosynthesis	98.0	95.0
*bauA*	Acinetobactin uptake	71.0	79.0
*bauB*	Acinetobactin uptake	100.0	98.0
*bauC*	Acinetobactin uptake	100.0	97.0
*bauD*	Acinetobactin uptake	100.0	99.0
*bauE*	Acinetobactin uptake	100.0	99.0
*bauF*	Acinetobactin-associated iron acquisition	100.0	99.0

## Data Availability

The original contributions presented in this study are included in the article/Supplementary Material. Further inquiries can be directed to the corresponding author.

## Supplementary Materials

The following supporting information can be downloaded at: https://www.mdpi.com/article/10.3390/microorganisms14091971/s1, Figure S1: Temporal distribution of mobilome content in global *Acinetobacter baumannii* isolates. Scatter plot showing the relationship between year of isolation and the number of mobile genetic elements (MGEs) in global isolates; Figure S2: Temporal analysis of mobilome content in Middle Eastern *Acinetobacter baumannii* isolates. Scatter plot showing the relationship between year of isolation and mobile genetic element (MGE) abundance among Middle Eastern isolates collected between 2008 and 2016; Figure S3: Temporal analysis of antimicrobial resistance gene burden in Middle Eastern *Acinetobacter baumannii* isolates (2008–2016).

## Abbreviations

The following abbreviations are used in this manuscript:

*A. baumannii*	*Acinetobacter baumannii*
AMR	Antimicrobial Resistance
CARD	Comprehensive Antibiotic Resistance Database
CDS	Coding Sequence
CLI	Command-Line Interface
CRISPR	Clustered Regularly Interspaced Short Palindromic Repeats
DNA	Deoxyribonucleic Acid
GTR	General Time Reversible (substitution model)
GWAS	Genome-Wide Association Study
HGT	Horizontal Gene Transfer
IC2	International Clone II
iTOL	Interactive Tree Of Life
*ISAba1*	Insertion Sequence Aba1
MGE	Mobile Genetic Element
ML	Maximum Likelihood
MLST	Multilocus Sequence Typing
MSA	Multiple Sequence Alignment
MDR	Multidrug-Resistant
Mb	Megabase
NCBI	National Center for Biotechnology Information
OR	Odds Ratio
PCA	Principal Component Analysis
rRNA	Ribosomal RNA
SD	Standard Deviation
tRNA	Transfer RNA
VFDB	Virulence Factor Database
XDR	Extensively Drug-Resistant
