# Supplementary material for: Comparative Population Genomics and Machine Learning Reveal a Distinct Regional Genomic Fingerprint of Middle Eastern Acinetobacter baumannii
Source: Microorganisms. 2026 Sep 7;14(9):1971. doi: 10.3390/microorganisms14091971 (PMC13609248; doi:10.3390/microorganisms14091971)
Supplement: Supplementary file 1 [file microorganisms-14-01971-s001.zip › microorganisms-4492943-supplementary.pdf]

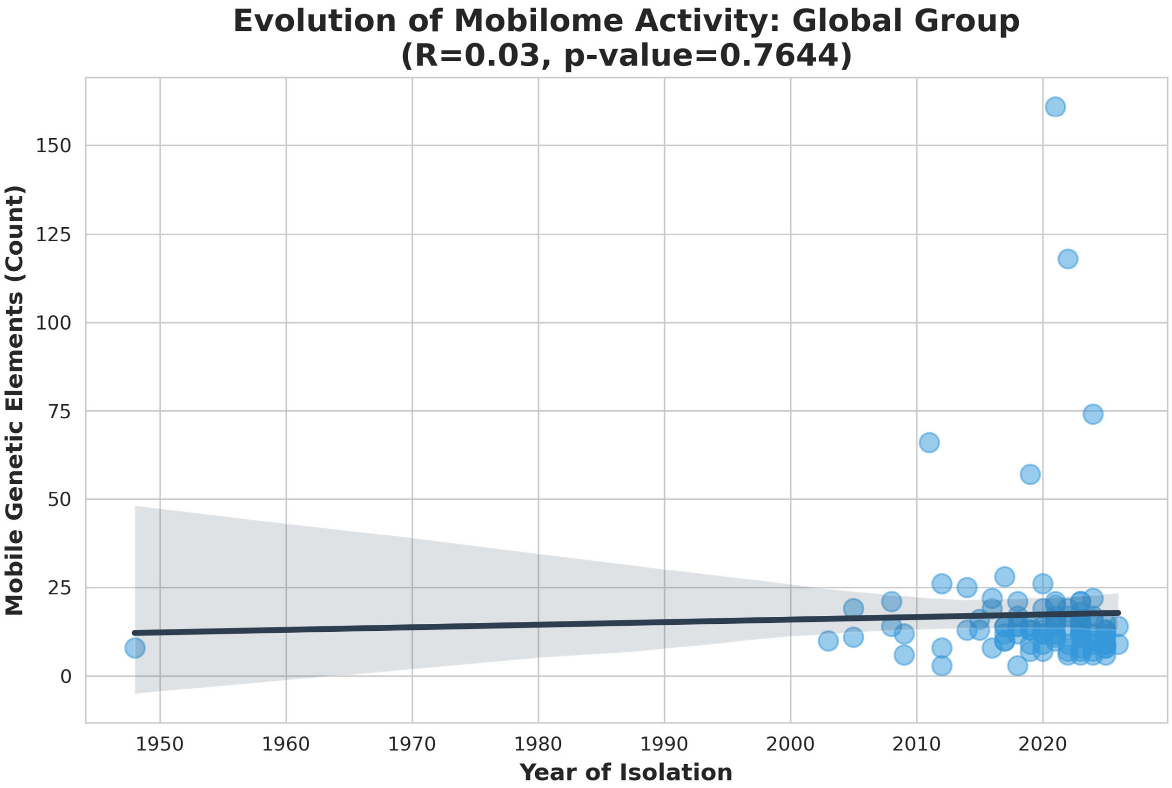

Supplementary Figure S1. Temporal distribution of mobilome content in global *Acinetobacter baumannii* isolates. Scatter plot showing the relationship between year of isolation and the number of mobile genetic elements (MGEs) in global isolates. Each point represents an individual genome, and the fitted regression line illustrates the overall temporal trend. No significant association was observed between isolation year and mobilome content (R = 0.03, p = 0.7644), indicating that MGE abundance remained largely stable over time despite the presence of several highly mobile outlier isolates.

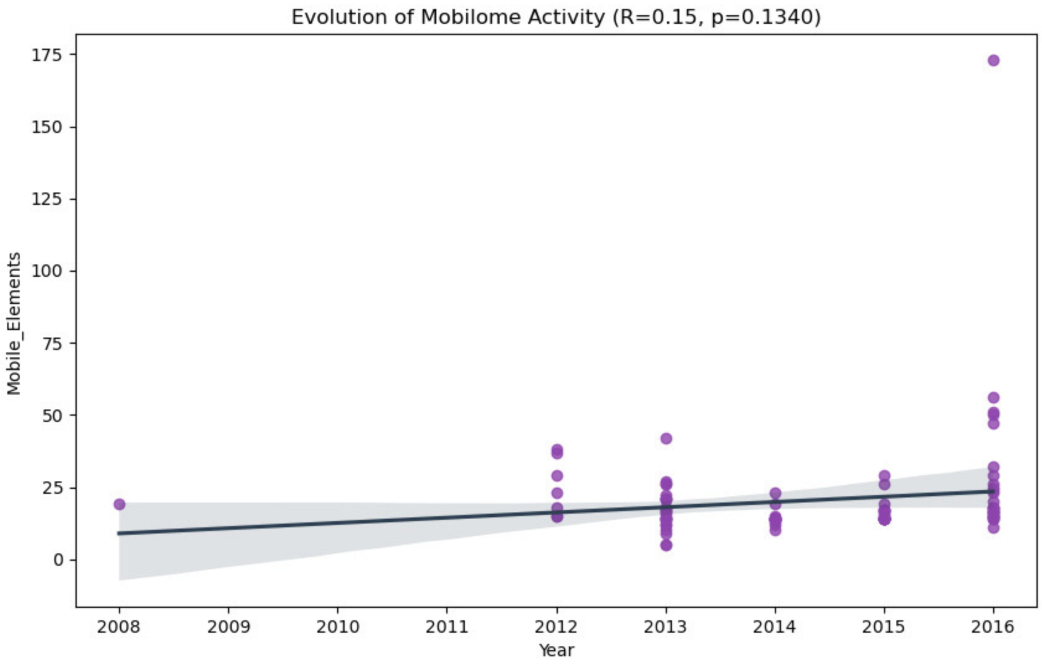

Supplementary Figure S2. Temporal analysis of mobilome content in Middle Eastern Acinetobacter baumannii isolates. Scatter plot showing the relationship between year of isolation and mobile genetic element (MGE) abundance among Middle Eastern isolates collected between 2008 and 2016. Each point represents an individual genome, and the regression line indicates the overall temporal trend. Although a weak positive correlation was observed (R = 0.15), the relationship was not statistically significant (p = 0.1340), suggesting that mobilome content remained relatively stable throughout the study period. Several isolates exhibited elevated MGE counts, but these did not substantially alter the overall temporal pattern.

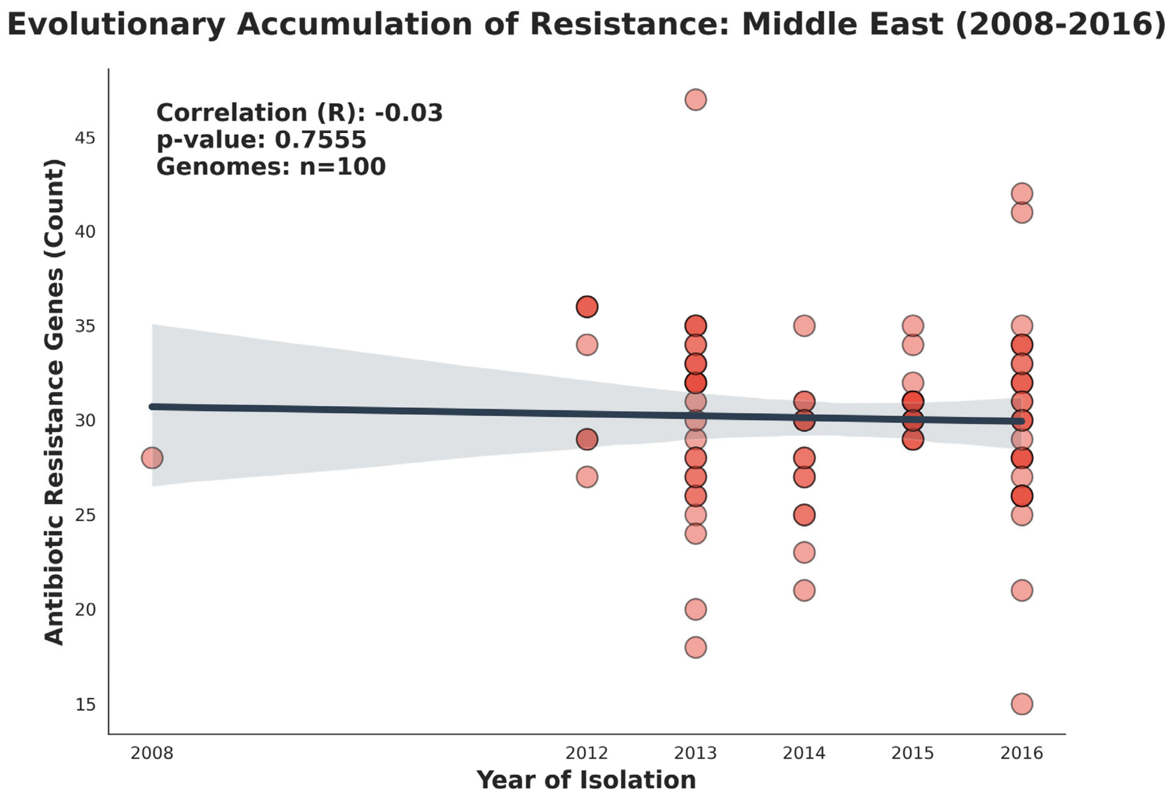

Supplementary Figure S3. Temporal analysis of antimicrobial resistance gene burden in Middle Eastern *Acinetobacter baumannii* isolates (2008–2016). Scatter plot showing the relationship between year of isolation and the number of antimicrobial resistance genes detected in Middle Eastern *Acinetobacter baumannii* genomes. Each point represents an individual isolate, and the fitted regression line illustrates the overall temporal trend. Resistance gene counts ranged from approximately 15 to 47 genes per genome, with most isolates harboring between 25 and 35 resistance determinants. Linear regression revealed a very weak negative correlation between isolation year and resistance gene burden ($R = -0.03$), which was not statistically significant ($p = 0.7555$). These findings indicate that resistance gene content remained largely stable throughout the study period, with no evidence of progressive accumulation or reduction in antimicrobial resistance determinants between 2008 and 2016.
